# Systematic Review of Sleep Disturbances and Circadian Sleep Desynchronization in Autism Spectrum Disorder: Toward an Integrative Model of a Self-Reinforcing Loop

**DOI:** 10.3389/fpsyt.2019.00366

**Published:** 2019-06-06

**Authors:** Claudia Carmassi, Laura Palagini, Danila Caruso, Isabella Masci, Lino Nobili, Antonio Vita, Liliana Dell’Osso

**Affiliations:** ^1^Department of Clinical and Experimental Medicine, Psychiatry Division, University of Pisa, Pisa, Italy; ^2^Child Neuropsychiatry Unit, IRCCS G. Gaslini Institute, Genova, Italy; ^3^Department of Neuroscience–Rehabilitation–Ophthalmology–Genetics–Child and Maternal Health (DINOGMI), University of Genova, Genova, Italy; ^4^Psychiatry Division, Department of Clinical and Experimental Medicine, University of Brescia, Brescia, Italy

**Keywords:** autism spectrum disorder, circadian rhythms, sleep disturbances, neurodevelopmental, melatonin, clock genes

## Abstract

**Background:** A compelling number of studies, conducted in both children and adults, have reported an association between sleep disturbances/circadian sleep alterations and autism spectrum disorder (ASD); however, the data are sparse and the nature of this link is still unclear. The present review aimed to systematically collect the literature data relevant on sleep disturbances and circadian sleep dysrhythmicity related to ASD across all ages and to provide an integrative theoretical framework of their association.

**Methods:** A systematic review of the MEDLINE, PubMed, and Cochrane databases was conducted from November 2018 to February 2019. The search strategies used were MeSH headings and keywords for “sleep–wake circadian rhythms” OR “circadian sleep disorders” OR “sleep–wake pattern” OR “sleep disorders” OR “melatonin” AND “autism spectrum disorder” OR “autism”.

**Results:** One hundred and three studies were identified, 15 regarded circadian sleep dysrhythmicity, 74 regarded sleep disturbances, and 17 regarded melatonin alterations in children and adults with ASD. Our findings suggested that autistic subjects frequently present sleep disturbances in particular short sleep duration, low sleep quality/efficiency, and circadian sleep desynchronization such as delayed phases and/or eveningness. Sleep disturbances and circadian sleep alterations have been related to the severity of autistic symptoms. Genetic studies have shown polymorphisms in circadian CLOCK genes and in genes involved in melatonin pathways in subjects with ASD.

**Conclusions:** Sleep disturbances and circadian sleep alterations are frequent in subjects with autistic symptoms. These subjects have shown polymorphisms in clock genes expression and in genes involved in melatonin production. The impairment of circadian sleep regulation may increase the individual’s vulnerability to develop symptoms of ASD by altering the sleep regulation *in toto*, which plays a key role in normal brain development. Even though controversies and “research gaps” are present in literature at this point, we may hypothesize a bidirectional relation between circadian sleep dysfunction and ASD. In particular, circadian sleep dysrhythmicity may predispose to develop ASD symptoms and vice versa within a self-reinforcing feedback loop. By targeting sleep disturbances and circadian sleep dysrhythmicity, we may improve treatment strategies for both children and adults with ASD.

## Highlights

- Children and adults with autistic symptoms often experience sleep disturbances and alterations in circadian sleep rhythmicity.
- Children and adults with autistic symptoms have shown mutations and polymorphisms in clock gene expression related to irregular and delayed sleep phases and to sleep problems.
- Abnormalities in the expression of genes regulating melatonin pathways may be responsible for low melatonin levels and for circadian sleep disturbances in subjects with autistic symptoms.
- Genetic abnormalities in the circadian system may impair the sleep system *in toto* with negative consequences on brain development contributing to autistic symptoms.
- The impairment of circadian sleep rhythmicity may increase the individual’s vulnerability to develop symptoms of autism spectrum disorder.
- Vice versa, alteration in brain development related to autism spectrum disorder autistic symptoms may contribute to sleep disturbances within a self-reinforcing loop.
- The evaluation and treatment of circadian sleep disorders in autism spectrum disorders may be useful to improve the trajectories of subjects with autistic symptoms.

## Introduction

Autism spectrum disorder (ASD) is an early-onset neurodevelopmental disorder whose core features have been defined by the *Diagnostic and Statistical Manual of Mental Disorders, Fifth Edition* (*DSM-5*) (1). It is characterized as persistent difficulties in social interaction and communication, presence of stereotypic behaviors, restricted interests, and atypical sensory reactivity. ASD, in fact, encompasses a set of clinical phenotypes that includes previously described autistic disorder (AD) and Asperger syndrome (AS), from severe to mild variants as endpoints of a continuum upon a spectrum model approach (2, 3). Intellectual disability is observed in more than half of ASD cases (4, 5) and ASD autistic symptoms also affect social language skills and emotion regulation. In addition, ASD symptoms are often related to coexisting mental disorders and to other developmental disorders (6).

Within the last decades, the diagnosis rate of autism has increased dramatically, and it has been reported that cases of ASD have a rate of 0.6–0.8% in preschool children, 1.0% in school children and young adults, and around 1.0% in adults (7, 8). In the last few years, an increased interest has been developed for mild forms of ASD, which often remain undiagnosed or misdiagnosed until adulthood. Although ASD is defined as a developmental disorder because symptoms appear within the first 2 years of life, it is generally considered a lifelong disorder with negative consequences on scholastic, working, social, and economic performances and quality of life. Some studies have shown that mild forms of autism are related to high rates of psychiatric comorbidity in adulthood such as anxiety, mood disorders, psychosis, stress-related disorders, and suicidal behaviors (9, 10).

In this framework, understanding the mechanisms involved in the development of ASD should be a priority for identifying early markers that could help improve early diagnosis with a significant impact on lifelong prognosis (3). The mechanisms underlying the development of ASD may be the result of the interaction between multiple gene arrangement and the environment that may lead to the alteration of brain structures and functions (11, 12). This interaction may determine epigenetic alterations disrupting the regulation of gene expression with a negative impact on biological pathways relevant for brain development (13). Abnormalities during brain development in autistic subjects go beyond the “social brain” encompassing sensory processing and attentional control. In fact, current evidence strongly supports a model of brain-wide abnormalities during the early development of autistic children (12). Indeed, it remains an open question if a single mechanism or several independent factors can lead to the emergence of autism.

In the last few years, a new hypothesis has emerged, suggesting the role of circadian system desynchronization in the development of ASD (11, 14). Human physiological and biochemical processes as well as behavioral patterns have a circadian rhythmicity orchestrated by the master biological clock of the hypothalamus: the suprachiasmatic nuclei (SCN). The expression of many genes changes rhythmically over 24 h and the specific circadian genes are responsible for the main SCN clock-working machinery as well as that of subsidiary clocks at the peripheral level [among them: circadian locomotor output cycles kaput (CLOCK), brain and muscle ARNT-like protein 1 (BMAL1), cryptochromes CRY1-2, and band period homolog (PER)] [(15, 16); for an overview see Ref. (17)]. The suprachiasmatic nuclei is daily synchronized by environmental signals such as light, food intake, activities, or social cues and exposure to stress/trauma (18–20), and while driving, secretion of the melatonin hormone regulates peripheral clock within feed-forward mechanism. Rhythmic clock gene expression regulates multiple monoaminergic brain regions that control mood and motivational behaviors, stress and inflammatory systems, reward circuits, arousal, and sleep by interacting with the homeostatic regulation of sleep and wake [for an overview, see Refs. (17, 21)]. The circadian system is critical for the synchronization with the environment and allows a correct functioning of various internal physiological processes essential for the optimization of responses to environmental fluctuations and for the strengthening of homeostatic control mechanisms (21). Abnormalities in the maturation of the circadian system principally lead to alterations in the sleep–wake pattern (22), which may interest around 50–80% of subjects with ADs (23). Wimpory and colleagues (24) have hypothesized that timing and social timing deficits were relevant in subjects with AD symptoms and were related to pathological variations in the structure/function of clock/clock-related genes: the authors hypothesized a key role for circadian sleep dysregulation in autistic symptoms. This hypothesis has been confirmed by later studies, which have shown circadian-relevant gene abnormalities in ADs (25, 26). Mutations in Clock, Bmal1, Cry1, and Cry2 genes determine an alteration in the circadian system regulation as well as in sleep fragmentation (27, 28). Sleep is increasingly recognized as a key process in neurodevelopment and in brain optimization processes [for an overview, see Ref. (29)]. Both humans and animals’ data have shown that sleep is essential for maturation of fundamental brain functions, and epidemiological findings increasingly indicate that children with early sleep disturbances suffer from later cognitive, attentional, and psychiatric problems. Indeed, from birth throughout infancy and early childhood, sleep patterns undergo dramatic changes that include the gradual consolidation of sleep and waking cycles, the intensification of deep NREM sleep slow-wave activity (EEG power in the 1–4.5 Hz frequency range), and a progressive decrease of REM sleep proportion. It has been suggested that REM sleep is an inducer of brain development and of early myelination in the sensory processing areas in the fetus and the newborns and that it follows the maturational trajectory of the brain (29–34). Animal studies have shown that REM sleep has multifaceted functions in brain development, including learning and memory consolidation by selectively eliminating and maintaining newly formed synapses (35). On the other hand, slow-wave sleep and sleep spindles (10–14 Hz) seem to be even involved in synaptic remodeling, being important for synaptic strength and synchronized neuronal firing, and it has been shown that while following the trajectory of brain maturation, they may orchestrate synaptic plasticity and pruning during brain development (30, 36–40). Particularly, Ringli and Huber hypothesized that slow-wave sleep may contribute to cortical maturation by playing a role in the balance of brain synaptic strengthening/formation weakening/elimination that is tilted during development (39). Sleep promotes myelination and oligodendrocyte precursor cell proliferation (41), enhances transcription of genes involved in synthesis and maintenance of membranes and myelin too (42), and modulates the neuronal membrane homeostasis (43). Since adequate sleep has been proposed to be fundamental for brain development (31, 35, 39), sleep has received considerable research attention, as it appears to be important in the study of neurodevelopmental psychopathology (31, 39, 44–46). Hence, if sleep is fundamental for brain development, we may hypothesize that sleep disturbances, *via* alterations in brain development, may contribute to autistic symptoms. This idea has been previously developed for other psychiatric disorders. In fact, extensive data have shown that poor sleep during childhood and adolescence is related to alterations in brain development (39, 44, 47–53) to problems in cognitive, attentional, emotional, and behavioral areas, including risk-taking and aggression; and to psychiatric conditions such as attention deficit hyperactivity disorder and mood disorders (54). Indeed, we may also hypothesize that alteration in brain development related to ASD may contribute to sleep disturbances within a self-reinforcing loop.

## Objectives

On this basis, this review was aimed to systematically collect the literature data relevant on sleep disturbances and circadian sleep dysrhythmicity related to ASD across all ages; a comprehensive review that has been conducted recently was limited to youth (55). In particular, we aimed to construct an integrative theoretical framework of their association: a comprehensive framework would be quite useful from a clinical and therapeutic point of view for identifying elements to evaluate and target in the clinical practice.

The hypothesis of this review was that abnormal maturation of the circadian sleep system may lead to the disruption of the sleep system *in toto* that, by impairing brain neurodevelopment and melatonin production, may have a key role in ASD. The investigation of such elements may be helpful to a better understanding of the neurodevelopmental pathways of ASD. Hence, the aim of this review was to systematically collect the literature data on sleep and circadian disturbances in children and adults with ASD.

## Methods

### Search Strategy

We performed a systematic review, based on Preferred Reporting Items for Systematic Review and Meta-analyses (PRISMA) guidelines, of articles published up till February 2019, and indexed in the following databases: MEDLINE, PubMed, and Cochrane Library. The search strategies used MeSH headings and keywords for “sleep–wake circadian rhythms” OR “circadian sleep disorders” OR “sleep–wake pattern” OR “sleep disorders” OR “melatonin” AND “autism spectrum disorder” OR “autism.”

*Inclusion criteria:* The searches were limited to the English language studies conducted on human populations, including longitudinal, cross-sectional, or case–control studies, analyzing the relationship between sleep disturbances and/or circadian sleep disorders and ASD.

*Exclusion criteria:* Studies that did not have as principal focus the evaluation of sleep disturbances and/or circadian sleep rhythms related to autism were excluded. We also excluded studies that investigated subjects with other neurodevelopmental disorders or intellectual disabilities.

### Data Extraction

**Figure 1:**
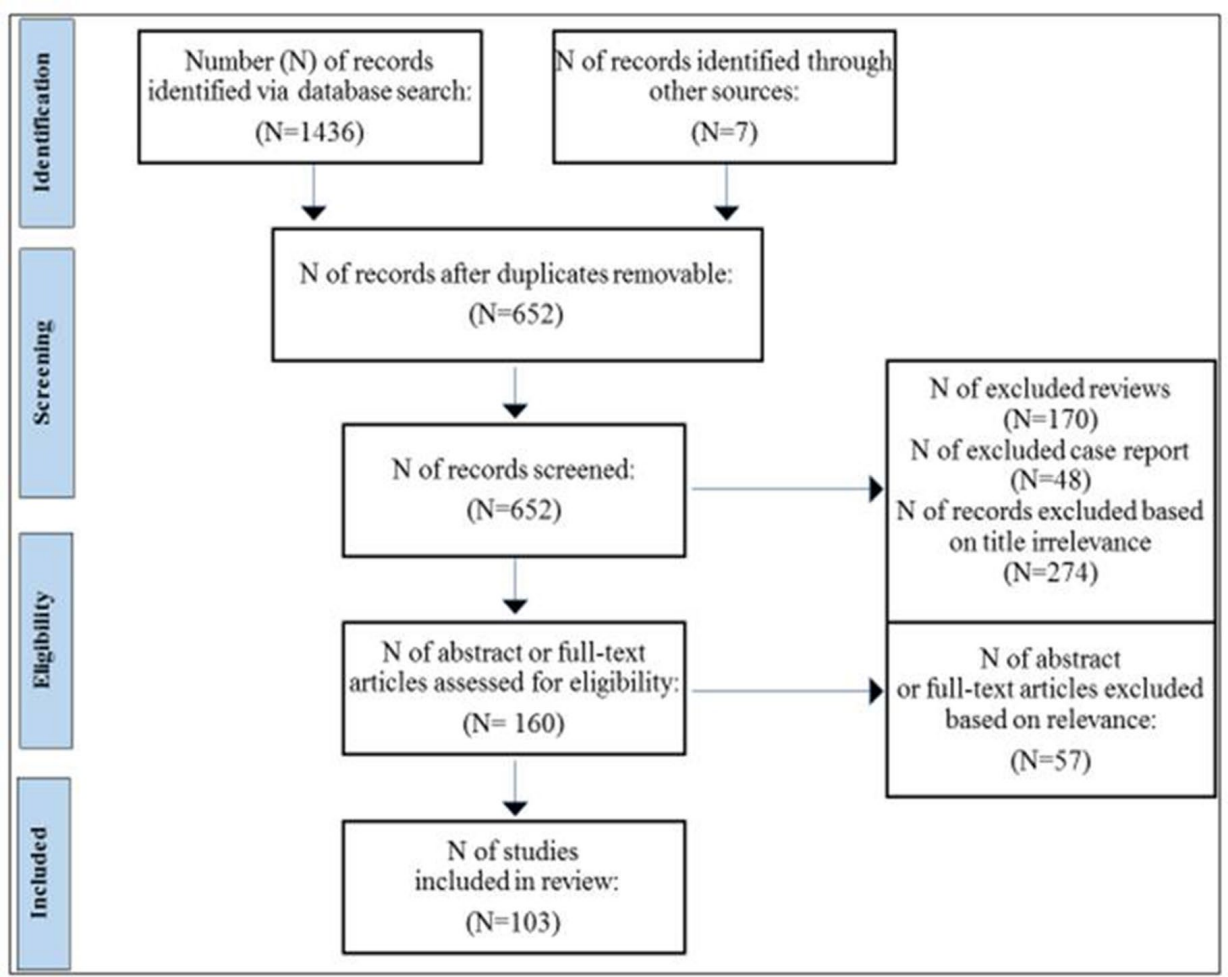
Flow chart based on PRISMA guidelines.

The abstracts located from the search strategy were entered into EndNote. The searches were limited to the English language studies conducted on human populations, including longitudinal, cross-sectional, or case–control studies, analyzing the relationship between sleep disturbances and/or circadian sleep disorders and ASD. Title and abstracts of all non-duplicated papers were independently screened by two of the authors (DC and IM). Potential pertinent papers were retained and assessed for eligibility by screening the full text. Two senior authors (LP and CC) acted as arbitrators when there is disagreement in any screening stage. All the authors independently evaluated the quality of the included studies. Disagreements between authors were resolved by consensus. Initial literature search returned 1,443 records (among which 7 studies were searched manually from other sources), 652 after exclusion of duplicates. Following preliminary screening of the titles and the exclusion of reviews and case reports, 492 of the retrieved articles were excluded; further 57 records were excluded after reading abstracts and full texts based on the relevance of data for the aim of the present study (**Figure 1**).

### Data Analysis

Meta-analysis was not conducted due to heterogeneity in definition and measurement of outcomes.

## Results

The systematic review of literature returned 103 articles focused on the sleep–wake pattern in neurodevelopmental disorders on children and an adult population according to the evolution of nosography; these studies include both previously defined diagnoses (56), such as AD, AS, childhood disintegrative disorder (CDD), pervasive developmental disorder (PDD), and not otherwise specified (NAS) (2), and the most recent diagnosis of ASD (1).

### Circadian Sleep Dysrhythmicity in Children With ASD

Seven studies have evaluated the alterations of circadian rhythms in children with ASD (**Table 1**). The first study (57) reported no tendency for irregular sleep–wake pattern, delayed sleep phase syndrome, or advanced sleep phase syndrome in children with ASD. Similar results were found in another study and in addition, no circadian phase preference was detected (61). On the other hand, several studies have shown that children with ASD may show sleeplessness with two specific circadian problems: phase delay of sleep periods and an irregular sleep–wake pattern. Some recent studies have investigated the genetic patterns of circadian rhythmicity in autistic subjects. First, Nicholas et al. (25) screened 110 individuals with ASD and their parents, analyzing the single-nucleotide polymorphisms (SNPs) in 11 clock/clock-related genes. A significant allelic association was detected for Period Circadian Regulator 1 (PER1) and Neuronal PAS domain protein 2 (NPAS2) and ASD. A later study (59) described the circadian deficits in del 2q231 of lymphoblastoid cell lines (LCLs) in 19 ASD children with high prevalence of sleep disturbances. They found that circadian gene mRNA levels of NR1D2 (nuclear receptor subfamily 1 group D member 1), PER1, PER2, and PER3 were altered in del 2q23.1. Yang et al. (26) sequenced the coding regions of 18 canonical clock genes and clock-controlled genes in 28 ASD patients and 23 controls. They detected several mutations/SNPs in circadian-relevant genes affecting gene function in the ASD patients. Mutations in NR1D1, CLOCK, and ARNTL2 were detected only in individuals with ASD with sleep disorder. The most recent work (60) tested mutations in all exons of NR1D1 in 198 autistic subjects. They detected single-base changes with an amino acid substitution in the coding region of NR1D1 in individuals with ASD. In summary, children with ASD show circadian and sleep rhythms alterations such as irregular and delayed sleep phase and several mutations in the expression of clock genes.

**Table 1:** Clinical studies on circadian rhythmicity and autism spectrum disorder in children.

Authors (Ref.) country	Study design	Sample size	ASD assessment	Sleep–wake cycle assessment	Results	Major limitations
Takase, Taira and Sasaki (57) Japan	Cross-sectional study	89 autistic children (3–20 years).	Clinical diagnoses.	Actigraphy Total sleep time (TST) was calculated from their sleep logs.	One autistic girl showed a tendency of non-24-h sleep–wake syndrome. The others did not. Most subjects showed a large variation in TST.	No diagnostic criteria for diagnosis, no control group.
Nicholas et al. (25) Autism Genetic Resource Exchange	Cross-sectional study	110 autistic subjects and their parents.	ADI-R, ADOS-G94, *DSM-IV* diagnostic criteria.	Analysis of the single-nucleotide polymorphisms (SNPs) in 11 clock/clock-related genes.	A significant allelic association was detected for PER1 and NPAS2.	Predominant high-functioning subjects.
Giannotti et al. (58) Rome, Italy	Case–control study	104 children with autism; 162 TD children.	*DSM-IV-TR* diagnostic criteria; ADI-R; ADOS-G; CARS.	CSHQ; Parental report sleep diary for 4 weeks; 21 channel EEG recordings.	Regressed group showed higher incidence of circadian rhythm disorders than non-regressed ones. The regressed group showed higher CSHQ bedtime resistance, sleep onset delay, sleep duration and night awakening scores.	No evaluation of sleep parameters by standardized measures.
Mullegama et al. (59) Richmond, VA, USA	Cross-sectional study	19 children with a molecular diagnosis of del 2q23.1 (9 months to 11 years).	Molecular diagnosis of del 2q23.1	Parent sleep questionnaire; The expression of four circadian genes, NR1D2, PER1, PER2, and PER3, in 2q23.1 deletion syndrome lymphoblastoid cell lines (LCLs).	Molecular analysis of the circadian deficits associated with haploinsufficiency of MBD5 in which circadian gene mRNA levels of NR1D2, PER1, PER2, and PER3 were altered in del 2q23.1 of LCLs; haploinsufficiency of MBD5 can result in dysregulation of circadian rhythm gene expression. Circadian and mTOR signaling pathways were associated with sleep disturbance.	Small sample size; no objective sleep measures.
Yang et al. (26) Tochigi, Japan	Case–control study	28 ASD patients 23 controls of Japanese descent.	*DSM IV-TR* diagnostic criteria	The coding regions of 18 canonical clock genes and clock-controlled genes were sequenced.	The mutations p.S20R in NR1D1, p.H542R in CLOCK, p.L473S in ARNTL2, p.A325T in TIMELESS, p.S13T in ARNTL, and p.G24E in PER2 were diagnosed in ASD. Mutations in circadian-relevant genes affecting gene function were more frequent in patients with ASD than in controls.	Small sample size; no objective sleep measures.
Goto et al. (60) Nagoya, Japan	Case–control study	111 Caucasian 87 Japanese patients with ASD; 158 Caucasian and 133 Japanese TD children.	*DSM IV-TR* and *DSM-5* diagnostic criteria.	The patients, their siblings, and parents were tested for mutations in all exons of NR1D1 (also known as Rev-Erbα).	They detected single-base changes with an amino acid substitution in the coding region of NR1D1 in 4 individuals. Not detected in controls. c.1012C were identified as the rare SNPs. A (p.R500H) mutation (AU1098302) had typical features of ASD and no difficulty in sleep induction; he showed strong anxiety and little sociability without verbal communication with others.	No objective sleep measures.
Van der Heijden et al. (61) The Netherlands	Case–control study	44 children with ADHD; 67 children with ASD; 243 TD children (8–12 years).	Parent report CBCL.	Sleep Disturbance Scale for Children parental report questionnaire; Chronotype of the children was assessed with Children’s Chronotype Questionnaire; Sleep hygiene was assessed using the Children’s Sleep Hygiene Scale	Children with ADHD and ASD showed more sleep problems (63.6 and 64.7%, vs. 25.1% in TD) and shorter sleep duration than controls, while differences between ADHD and ASD were not significant. Evening types were associated with sleep problems in ADHD and ASD. Associations of greater anxiety/depression with sleep problems were shown in ADHD and TD.	No objective sleep measures.

DSM, Diagnostic and Statistical Manual; TD, typically development; NDD, neurodevelopment disorder; CSHQ, Children’s Sleep Habits Questionnaire; ICD-10, International Classification of Diseases-10; ASD, autism spectrum disorder; ADI_R, Autism Diagnostic Interview-Revised; ADOS, Autism Diagnostic Observation Schedules; PSG, polysomnography; CBCL, child behavior checklist; ADHD, attention-deficit hyperactivity disorder; PER1, Period Circadian Regulator 1; NPAS2, Neuronal PAS domain protein 2; NR1D1-2, nuclear receptor subfamily 1 group D member 1–2; MBD5, methyl-CpG binding domain protein 5.

### Sleep Disturbances in Children With Autism Spectrum Disorder

Sixty-five studies have been evaluated on the sleep pattern of children with ASD (**Table 2**), and some of them reported a prevalence rate of sleep disturbances between 64% and 93% (65, 71, 95, 108, 109, 114, 125) underlining a great prevalence of sleep disturbances during early life (62, 63, 65, 86, 109, 125) with no gender differences (62, 89). In particular, total sleep time has shown to be reduced starting from 30 months of age toward adolescence (98, 104, 107).

**Table 2:** Clinical studies on sleep disturbances in autism spectrum disorder in children

Authors (Ref.) country	Study design	Sample size	ASD assessment	Sleep–wake cycle assessment	Results	Major limitations
Hoshino et al. (62) Fukushima Japan	Case–control study	75 children with autism (3–15 years); 75 TD children (3–11 years).	WHO and Kanner’s diagnostic criteria; Infantile Developmental Test.	Sleep pattern was checked by the parents day-to-day for 1 month.	65% of autistic children showed sleep disturbance, no gender differences. Poorly developed autistic children showed a higher rate of sleep disturbance vs. well-developed autistic children.	No psychometric assessment of sleep pattern.
Richdale and Prior (63) Australia	Case–control study	39 children with autism (32 months–19 years): IQ < 55 (low-functioning, *N* = 12) vs. IQ > 55 (higher-functioning, *N* = 27); 35 TD children.	*DSM-III* diagnostic criteria. IQ: Leiter International Performance Scale or Bayley Infant Development Scales or Mertill-Palmer Scale of Mental Tests.	Parents completed 14-day sleep diaries and questionnaires.	During childhood, most of the children with autism experienced sleep problems: extreme sleep latencies, lengthy periods of night awakening, shortened night sleep, and early morning waking.	Small sample size; no evaluation of sleep parameters with standardized assessment.
Patzold et al. (64) Australia	Case–control study	31 children with autism; 36 TD children.	*DSM-III* or *DSM-III-R* diagnostic criteria.	A sleep diary completed by parents over a 2-week period; behaviors questionnaires.	Children with autism were likely to fall asleep later at night, have longer sleep latencies, sleep less at night, and spend a significant period of time awake during the night, compared with control group.	Small sample size; No evaluation of sleep parameters by standardized assessment.
Taira, Takase and Sasaki (65) Japan	Cross-sectional study	88 children with autism.	Clinical diagnosis.	Sleep questionnaires.	Sleep disorders were observed in 56 children, 44 of whom had sleep disorders before 3 years old. The most common problem was difficulty falling asleep, frequent awakening during sleep time, early morning awakening.	No control group; no evaluation of sleep and autism with standardized assessment.
Diomedi et al. (66) Roma, Italy	Cross sectional study	10 mentally retarded autistic subjects (12–24 years); 8 Down syndrome subjects; 8 TD subjects.	DSM-IV diagnostic criteria; CARS; Psychological Educational Profile (PEP).	Two consecutive overnight PSG.	Compared to normal subjects, autistic subjects presented a significant reduction of REM %, a fragmentation of REM periods, due to a frequent intrusion of NREM 1 and 2, an increased number of awakenings with a consequent reduction of Sleep Efficiency.	Small sample size.
Hering et al. (67) Israel	Comparative study	22 children with autism: 12 with sleep problems	Clinical diagnosis.	Sleep questionnaire; 72 h of actigraphy.	Autistic children showed early morning awakenings and multiple and night arousals	Small sample size; no evaluation of autism parameters by standardized assessment.
Godbout et al. (68) Canada	Case–control study	8 patients with AS (7–53 years); 8 TD age-/sex-matched subjects.	*DSM-IV* diagnostic criteria; ADI.	Sleep was recorded for two consecutive nights.	Patients with AS showed decreased sleep time in the first 2/3 of the night, increased number of shifts into REM sleep from waking, and REM sleep disruption; sleep spindles were significantly decreased.	Small sample size; use of medications.
Elia et al. (69) Italy	Comparative study	17 children and adolescents with ASD (5–16 years); 7 patients with mental retardation and fragile X syndrome; 5 TD subjects.	*DSM-IV* diagnostic criteria; karyotyping; neurometabolic screenings; brain imaging; Psychoeducational Profile-revised test (PEP-R); CARS.	Two overnight PSG with one adaptation night.	Density of REM was not significantly different in the three groups; some sleep parameters such as time in bed, and total sleep time were significantly lower in ASD subjects than in TD ones; CARS scores to visual response and nonverbal communication showed significant correlation with some sleep parameters.	Small sample size.
Honomichl et al. (70) California	Longitudinal study	100 children with PDD	Children were diagnosed with ASD, PDD not otherwise specified, AS, or other related disorders.	Two sleep diary data collection periods; CSHQ; Parenting Events Questionnaire.	All children with PDD exhibited longer sleep onset and greater fragmentation of sleep than that reported for age-matched community norms.	
Wiggs and Stores (71) Oxford, UK	Cross-sectional study	69 children with ASD (aged 5 to 16 years).	ICD-10 diagnostic criteria.	Sleep histories from parents; Simonds and Parraga Sleep Questionnaire; 2-week sleep diary; actigraphy for five nights.	Parent-reported sleeplessness (64%). Sleep disorders underlying the sleeplessness were most commonly behavioral; sleep patterns measured objectively did not differ between those children with or without reported sleeplessness, but the sleep quality of all children seemed to be compromised compared with normal values.	Sample included children of various ages and intellectual levels.
Gail Williams, P et al. (72) England	Cross-sectional study	210 children with autism.	*DSM-IV* diagnostic criteria; Wechsler Intelligence Scale for Children, 3ed; Differential Ability Scales Mental Retardation (MR).	Likert-based questionnaire for parent report.	Sleep problems reported: difficulty in falling asleep, restless sleep, not falling asleep in own bed, and frequent wakings, sleepwalking, morning headaches, crying during sleep, apnea, and nightmares. No significant differences were identified in frequencies of reported sleep problems between MR and not MR groups.	No evaluation of sleep parameters by objective measures.
Schreck et al. (73) USA	Cross-sectional study	55 children with autism (5–12 years).	Gilliam Autism Rating Scale (GARS).	Parental report sleep questionnaire.	Children with strong responses to the environment at night and who tend to awaken at night show more markedly autistic-type communication patterns on the GARS.	No evaluation of sleep parameters by objective measures.
Polimeni et al. (74) Australia	Cross-sectional study	53 children with autism; 53 children with AS; 66 TD children.	Clinical diagnosis.	The Behavioral Evaluation of Disorders of Sleep (BEDS).	High prevalence of sleep problems with significantly more problems reported in the autism and AS groups.	No evaluation of sleep and autism parameters by standardized measures; use of medication.
Cotton & Richdale (75) Australia	Case–control study	153 children: 98 had an ID, 37 with autism, 15 with Down syndrome, 29 with Prader–Willi syndrome, and 29 with intellectual disability; 55 TD subjects.	Clinical diagnosis.	Parental report sleep questionnaire.	Sleep problems were more prevalent in autism than the other disorders. Sleep maintenance problems were common in autism.	Small sample size; no evaluation of sleep and autism parameters by standardized measures.
Allik et al. (76) Stockholm, Sweden	Case–control study	32 school-age children with AS and HFA; 32 TD age- and gender-matched children.	Clinical diagnosis; ASSQ.	Parent-Reported Sleep Problems; 1-week parent recorded Child Sleep Diary; 1-week actigraphy.	Parental report, sleep diary, and actigraphy showed that children in the AS/HFA group spent longer time awake in bed before falling asleep than the control group.	Small sample size; use of medication.
Allik et al. (77) Stockholm, Sweden	Case–control study	32 school-age children with AS and HFA; 32 TD age-/gender-matched children	Clinical diagnosis; ASSQ; Strengths and Difficulties Questionnaire.	Parent-reported sleep problems; 1-week parent-recorded Child Sleep Diary; 1-week actigraphy.	Parent-reported difficulties initiating sleep and daytime sleepiness were more common in children with AS/HFA than in controls. Children with insomnia showed more parent-reported autistic and emotional symptoms, and more teacher-reported emotional and hyperactivity symptoms than those without insomnia.	Small sample size; use of medication.
Krakowiak et al. (78) California	Case–control study	303 children with ASD; 63 children with other developmental delays (DD); 163 TD children. The mean age was 3.6 years.	ADI-R; ADOS. Cognitive and adaptive functioning was assessed with specific scales.	Parent-administered questionnaire.	53% of children with ASD showed at least one sleep problem, followed by 46% of children with DD, and 32% of the TD group. Children with ASD had higher problems with sleep onset and higher night awakening compared to the TD group. Sleep disturbances of ASD group were not associated with cognitive and⁄or adaptive delays.	No evaluation of sleep parameters by standardized measures.
Liu et al. (79) USA	Comparative study	108 children with ASD, 27 children with AS; 32 with other diagnoses of ASD.	Parental report diagnosis of ASD; ADOS-G.	CSHQ; A structured sleep and family demographic questionnaire.	86% of children had at least one sleep problem almost every day, including 54% with bedtime resistance, 56% with insomnia, 53% with parasomnias, 25% with sleep disordered breathing, 45% with morning rise problems, and 31% with daytime sleepiness. Individual sleep problems: restless during sleep (28.7%), difficulty falling asleep (28.1%), awakened by others in the morning (26.9%), bed-wetting (26.3%), and poor appetite in the morning (25.7%).	Lack of a control group.
Malow et al. (80) Nashville, TN	Cross-sectional study	21 children with ASD; 10 TD children (4–10 years).	*DSM-IV-TR* diagnostic criteria; Clinical diagnosis; ADOS; CBCL; Peabody Picture Vocabulary Test (PPVT).	CSHQ; sleep histories; 1-week sleep diaries; 2 consecutive nights of video monitoring combined with EEG and PSG.	Poor sleepers showed prolonged sleep latency and decreased sleep efficiency on night 1 of PSG and differed on insomnia-related subscales of the CSHQ (increased sleep onset delay and decreased sleep duration) and also affective and reciprocal social problems on the CBCL and the ADOS, respectively.	Small sample size.
Miano et al. (81) Italy	Case–control study	31 patients with ASD: 17 with I.Q. 25–40 4 with I.Q. 40–55 (3.7–19 years); 893 TD children and adolescents.	*DSM-IV* diagnostic criteria; CARS; Wechsler Intelligence Scale for Children Revised or Weschsler Adult Intelligence Scale.	Parental report sleep questionnaire; PSG (16 patients and 18 controls).	ASD children showed high prevalence of initiating and maintaining sleep problems, enuresis, repetitive behavior when falling asleep, daytime sleepiness. PSG: ASD children showed reduced time in bed, total sleep time, sleep period time and REM latency.	
Bruni et al. (82) Rome, Italy	Cross-sectional study	8 children with AS; 10 children with autism, 12 TD children.	ADOS; CBCL; Wechsler Intelligence Scale for Children—Third Edition Revised-WISC-III	Sleep Questionnaire; Pediatric Daytime Sleepiness Scale; PSG	AS children showed high prevalence of initiating and maintaining sleep problems and daytime sleepiness. Subjects with AS showed increased CAP rate in SWS and A1 percentage. In subjects with AS, verbal IQ had a significant positive correlation with total CAP rate and CAP rate in SWS and with global and SWS A1 index. The percentage of A2 negatively correlated with full-scale IQ, verbal and performance IQ. CBCL total score correlated positively with CAP rate and A1 index while externalizing score correlated negatively with A3%.	Small sample size.
DeVincent et al. (83) New York	Case–control study	112 children with PDD (49 with autistic disorder, 13 with AS, and 50 with PDD–not otherwise specified); 497 TD children.	*DSM-IV* diagnostic criteria; Early Childhood Inventory–4; ADOS.	Early Childhood Inventory–4.	18% of children with PDD met the criteria for sleep disturbance. There were no significant differences between PDD subtypes in either the rate or severity of sleep problem. Sleep-disturbed children in both samples exhibited more severe behavioral difficulties than children without sleep problems.	No evaluation of sleep parameters by objective measures.
Dominick et al. (84) USA	Retrospective study	39 children with a history of language impairment (HLI); 67 children with ASD.	*DSM-IV* diagnostic criteria; ADI-R; ADOS-G.	The Atypical Behavior Patterns Questionnaire-parental reported.	Over 2/3 of the children with autism experienced atypical patterns of sleep. Initial insomnia and middle insomnia each occurred in >50% of the children with ASD. 12% of the sleep-disturbed children with ASD have terminal insomnia. The presence of sleep disturbances in children with autism and with HLI combined was significantly related to the presence of depression.	Use of retrospective questionnaire; interviewers were not blind to the participant’s diagnosis; sample was not chosen at random.
Allik et al. (85) Stockholm, Sweden	Case–control study	16 school-age children with AS and HFA; 16 TD age-/gender-matched children.	Clinical diagnosis.	1-week actigraphy.	At follow-up (2–3 years after the baseline), children with AS/HFA showed longer night waking and lower sleep efficiency during weekends than the controls.	Small sample size; use of medication.
Goodlin-Jones et al. (86) California	Comparative study	68 children with autism; 57 children with developmental delay without autism (DD); 69 TD children (2.0–5.5 years).	ADOS; Test of cognitive ability and of adaptive functioning; ADI-R.	Actigraphy; sleep diary; CSHQ.	DD group after sleep onset exhibited more and longer awakenings than the other two groups. Autistic children exhibited less total sleep time/24 h than the other two groups. Parent reports of sleep problems were higher in the AUT and DD groups than the TD group, but parent reports did not concur with more objective measures for behavioral insomnia.	Results may not generalize to a more heterogeneous population-based, community sample or to children referred for a clinical sleep disorder. The CSHQ has not been validated for these ages.
Goodlin-Jones et al. (87) California	Comparative study	68 children with autism; 57 children with developmental delay without autism [DD]; 69 TD children (2.0–5.5 years).	ADOS; Test of cognitive ability and of adaptive functioning; ADI-R.	Children were studied on three occasions, separated by a 3-month interval. At each assessment: actigraphy for 1 week; sleep–wake diaries; CSHQ.	Both neurodevelopmental groups showed more sleep problem by actigraphy and the CSHQ than TD children. Sleep onset insomnia and night awakenings decreased respectively by 40% and 30% every 3-month periods of actigraphic records.	
Goodlin-Jones et al. (88) California	Comparative study	68 children with autism, 57 children with developmental delay without autism [DD]; 69 TD children (2.0–5.5 years)	Mullen Scales of Early Learning; Vineland Adaptive Behavior Scale; ADOS; ADI-R; Social Communication Questionnaire.	Actigraphy; sleep diary; CSHQ.	CSHQ was clinically useful for screening of sleep problems in TD young children as well as in children with diverse neurodevelopmental diagnoses; sleep problems were prevalent in young children.	
Paavonen J et al. (89) Finland	Case–control study	52 children with AS; 61 TD subjects (5–17 years).	*DSM-IV* or ICD-10 diagnostic criteria; CBCL; ASSQ.	Sleep Self-Report.	Problems with sleep onset and maintenance, sleep-related fears, negative attitudes toward sleeping, and daytime somnolence were more frequent in AS vs. TD. Short sleep duration was almost twofold (59% vs. 32%) in AS vs. TD; the risk for sleep onset problems was fivefold (53% vs. 10%) more common in AS vs. TD.	No evaluation of sleep parameters by standardized measures.
Souders et al. (90) Philadelphia, PA	Descriptive cross-sectional study.	59 children with ASD (26 with autism, 21 with PDD-NOS, and 12 with AS) (4–10 years); 40 TD subjects.	*DSM-IV-TR* diagnostic criteria; ADOS; Children enrolled as TD were screened with Social Communication Questionnaire (SCQ) and developmental history.	CSHQ; 17-day parental report sleep diaries; 10-nights of actigraphy.	66.1% of parents of children with ASD and 45% of parents of the control group reported sleep problems in their sons; 66.7% of children with ASD (75% autism, 52.4% PDD-NOS, 75% AS) and 45.9% of the control subjects had disturbed sleep by actigraphy.	No evaluation of sleep parameters by standardized measures; small sample size
Goldman et al. (91) Nashville, TN	Case–control study	42 children with ASD without intellectual disability (4 to 10 years); 16 age-compared TD children.	*DSM-IV-TR* diagnostic criteria ADOS; Repetitive Behavior Scales–Revised (RBS–R); Peabody Picture Vocabulary Test–III (PPVT–III).	CSHQ; Parental Concerns Questionnaire (PCQ); CBCL; Actigraphy: PSG.	ASD poor sleepers differed from ASD good sleepers on actigraphy (sleep latency, sleep efficiency, fragmentation) and PSG (sleep latency) measures, reporting inattention, hyperactivity, and restricted/repetitive behaviors. Fragmentation was correlated with more restricted/repetitive behaviors.	Parental measures to differentiate the poor and the good sleepers groups; small sample size.
Buckley et al. (92) New York	Comparative study	60 children with autism; 13 children with developmental delay; 15 children with TD (2–13 years)	ADOS; ADI-R.	CSHQ; PSG.	No differences between TD vs. developmental delay groups. Comparison of autistic children vs. TD children revealed short total sleep time, great slow-wave sleep percentage, and small REM sleep percentage (14.5% vs. 22.6%) in ASD ones.	Use of medications.
Giannotti et al. (93) Rome, Italy	Comparative study	22 children with non-regressive autism; 18 children with regressive autism; 12 TD children (5–10 years).	*DSM-IV TR* diagnostic criteria; ADI-R; ADOS-G; nonverbal IQ > 50 assessed by Leiter International Performance Scale.	CSHQ; an overnight PSG.	Regressed children reported high CSHQ score: bedtime resistance, sleep onset delay, sleep duration and night wakings. Regressive subjects had significantly less efficient sleep, less total sleep time, prolonged sleep latency, prolonged REM latency and more time awake after sleep onset than non-regressive and TD group.	Small sample size; no evaluation of sleep parameters by standardized measures; small sample size.
Anders et al. (94) Sacramento, CA	Case–control study	68 children with autism (ASD); 57 children with DD without ASD; 69 TD children.	ADOS; ADI–R.	Actigraphy for 7 consecutive days for each of the 3 recording weeks (initial evaluation, 3 months later, and again after 3 months) During each 7-day week, parents completed a daily sleep diary.	ASD group slept less per 24-h period and were less likely to awaken at night than children in the other two groups. Children in the DD group had more frequent and longer duration nighttime awakenings than children in the ASD group. Children in the two neurodevelopmentally disordered groups demonstrated more night-to-night variability in their sleep–wake measures than children in the TD group.	
Rzepecka et al. (95) UK	Cross-sectional study	187 children with ID and/or ASD.	ADOS; Developmental, Dimensional and Diagnostic Interview (3DI).	CSHQ; Spence Children’s Anxiety Scale-Parent Version (SCAS-P); Aberrant Behaviour Checklist-Community (ABC-C).	Significant positive correlations between sleep problems, challenging behaviors and anxiety in children with ID and/or ASD.	No evaluation of sleep parameters by standardized measures; small sample size
Schwichtenberg et al. (96) California	Comparative study	68 children with autism; 57 children with DD; 69 TD children (24–66 months).	ADOS; ADI-R; Mullen Scales of Early Learning (MSEL); General adaptive behavior scales.	7 consecutive 24-h periods of actigraphy; parent-report sleep–wake diary. Sleep was assessed for 7 consecutive days on 3 separate occasions over 6 months.	Children with autism napped less often and for shorter periods of time than children with DD Children with DD napped more like children in the TD group, who were 6 months younger. Each group displayed an expected shift in daytime sleep as more children matured out of their naps.	Lack of specific groups of intellectual disability.
Anders et al. (97) Sacramento, CA	Case–control study	68 children with autism (AUT); 57 children with DD without AUT; 69 TD children.	ADOS; ADI–R; Psychoeducational Profile—Revised (PEP-R); Bayley pegboard task; CBCL.	7 consecutive 24-h periods of actigraphy for each of the 3 recording weeks (initial evaluation, 3 months later and again after 3 months). During each 7-day week, parents completed a daily sleep diary.	Both autism and ID groups showed poorer daytime performance and behaviors than the TD one. These significant differences persisted over 6 months; long night awakenings and lower sleep efficiency predicted daytime sleepiness in the ID group; parent-report sleep problems were associated with daytime sleepiness and behavior problems.	No developmentally tests of daytime functioning valid for NDD and TD preschool-age children.
Goldman et al. (98) USA	Cross-sectional study	1,859 children of the Autism Treatment Network	*DSM-IV-R* diagnostic criteria; ADOS.	CSHQ; Parents Concerns Questionnaire.	Adolescents reported delayed sleep onset, short sleep duration, and daytime sleepiness; while toddlers reported bedtime resistance, sleep anxiety, parasomnias, and night wakings.	No evaluation of sleep parameters by standardized measures.
Sikora et al. (99) Colorado	Cross-sectional study	1193 children with ASD.	General adaptive behavior scales; Survey Interview Form, Second Edition; CBCL.	CSHQ.	ASD group with sleep problems showed internalizing and externalizing behavior problems, and poor adaptive skill development. Children with moderate to severe sleep problems had greater behavior difficulties.	
Taylor et al. (100) USA	Cross-sectional study	335 children with autism or PDD-NOS (1–10 years).	General adaptive behavior scales.	Behavioral evaluation of sleep disorders.	Children who slept fewer hours per night had lower overall intelligence, verbal skills, overall adaptive functioning, daily living skills, socialization skills, and motor development. Children who slept fewer hours at night with waking during the night had more communication problems. Breathing-related sleep problems and fewer hours of sleep related most often to problems with perceptual tasks.	No evaluation of sleep parameters by objective measures.
Siversten et al. (101) Norway	Longitudinal study	3700 children, of whom 28 have ASD.	ASSQ.	Parent-reported sleep problems; Strengths and Difficulties Questionnaire (SDQ).	In the ASD group, the prevalence of chronic insomnia was more than 10 times higher compared to controls. ASD was a strong predictor of sleep problems and emotional and behavioral problems explained a large proportion of this association.	No evaluation of sleep parameters by objective measures; no measure of the severity of the sleep problems.
Baker et al. (102) Australia	Comparative study	34 adolescents with HFASD (14–17 years); 27 TD adolescents.	Social Communication Questionnaire.	7-day sleep diary; actigraphy in 55% of adolescents with HFASD; Sleep Habits Survey, adapted from the School Sleep Habits Survey; Pediatric daytime sleepiness scale; flinders fatigue scale.	Adolescents with HFASD were 3 times more likely to report a sleep problem than their TD peers (46.2% vs. 14.8%). Adolescents with HFASD had decreased sleep efficiency, and more fatigue compared with TD adolescents. While TD adolescents generally experienced one symptom of insomnia, adolescents with HFASD were likely to experience 2 or 3 symptoms of insomnia.	Small sample size.
Hollway et al. (103) USA	Cross-sectional study	1583 children in the Autism Treatment Network (2–17 years).	*DSM-IV* diagnostic criteria; ADOS;CBCL; Mullen Scales of Early Learning (MSEL); Stanford-Binet Intelligence Scale: Fifth Edition.	Short Sensory Profile; CSHQ.	Anxiety, autism symptoms severity, sensory sensitivities, and GI problems were associated with sleep disturbances. IQ positively predicted sleep disturbance and children with AS were more vulnerable than others.	No evaluation of sleep parameters by objective measures.
Humphreys et al. (104) UK	Cross-sectional study	30 children with classical childhood autism; 15 children with atypical autism; 23 children with AS.	Social Communication Disorders Checklist; Wechsler Intelligence Scale for Children 3rd edition (WISC-III).	Parental report sleep questionnaires.	From aged 30 months to 11 years old, children with ASD slept for 17–43 min less each day than contemporary controls. No significant difference in total sleep duration was found in infancy, but from 30 months of age, children with ASD slept less than their peers. Nighttime sleep duration was shortened by later bedtimes and earlier waking times.	Small sample size; absence of a control group.
Nadeau et al. (105) USA	Cross-sectional study	102 children and adolescents with ASD and comorbid anxiety disorders (7–16 years).	ADI-R; ADOS; Social Responsiveness Scale (SRS) measuring severity of autism spectrum symptoms; Anxiety Disorders Interview Schedule; Pediatric Anxiety Rating Scale; Multidimensional Anxiety Scale for Children–Parent; report measuring of anxiety symptoms.	CBCL.	The number of sleep-related problems endorsed directly associated with parent ratings of social deficits, internalizing and externalizing symptoms, and anxiety symptoms, as well as with clinician-rated anxiety symptoms.	No evaluation of sleep parameters by objective measures; absence of a control group.
Richdale et al. (106) Australia	Case–control study	27 adolescents with HFASD (14–17 years); 27 age-/sex-matched TD adolescents (14–17 years).	The Centre for Epidemiological studies Depression scale; the anxiety subscale of the Depression, Anxiety and Stress Scale.	7-day sleep/wake diary. 55% of HFASD adolescents and all TD adolescents wore an actigraphy concurrently with the sleep diary. Sleep Habits Survey; Chronic Sleep Reduction Questionnaire; Sleep Anticipatory Anxiety Questionnaire (SAAQ) to measure pre-sleep arousal.	Adolescents with HFASD had significantly higher scores for sleep arousal compared with TD adolescents, and poorer daytime functioning. There were significant correlations between sleep variables and psychopathology variables the HFASD group, than in the TD group.	Small sample size.
Hodge et al. (107) San Bernardino, CA	Comparative study	108 children with ASD; 108 TD children (3–18 years).	Clinical diagnoses; Autism Index; Gilliam Autism Rating Scale-2.	CSHQ.	Poor sleep quantity and quality in children with ASD, particularly children aged 6–9 years. The sleep problems of children with ASD were unlikely to diminish with age.	
Tudor et al. (108) USA	Cross-sectional study	62 children with ASD	Parent-reported diagnosis; Non-Communicating Children’s Pain Checklist–Revised.	CSHQ.	93% of the sample scored above 41 on the CSHQ. Pain predicted overall sleep disturbance and three specific sleep problems: sleep duration, parasomnias, and sleep-disordered breathing. These specific sleep problems were predicted by specific modalities of nonverbal pain communication.	Parental report of diagnosis and all data.
May et al. (109) Australia	Case–control study	46 children with ASD; 38 TD children (7–12 years).	*DSM-IV-TR* diagnostic criteria; Social Responsiveness Scale; Wechsler Intelligence Scale for Children-Fourth Edition; Spence Children’s Anxiety Scale.	CSHQ.	The ASD group had more sleep disturbance than the TD group. Sleep disturbance at baseline predicted later anxiety. Sleep disturbance decreased over the year in children with ASD, but not in TD children. Reduced sleep disturbance was associated with improved social ability.	No evaluation of sleep parameters by objective measures; only high-functioning children analyzed.
Mazurek & Petrosky (110) USA	Cross-sectional study	1347 children and adolescents enrolled in the Autism Speaks Autism Treatment Network.	ADOS; CBCL; Short Sensory Profile.	CSHQ.	Anxiety was associated with all types of sleep problems (bedtime resistance, sleep-onset delay, short sleep duration, sleep anxiety, and night waking). Sensory responsivity was correlated with sleep problem, but it was not significantly associated with bedtime resistance or sleep anxiety for younger children.	No evaluation of sleep parameters by objective measures; absence of a control group.
Wang et al. (111)	Cross-sectional study	60 Chinese children with ASD (6–17 years).	*DSM-IV-TR* diagnostic criteria.	CSHQ; Strengths and Difficulties Questionnaire.	Sleep disturbances were severe and common, with rates of 70.0% for overall disturbances and 15.0% (daytime sleepiness) to 40.0% (sleep duration) for specific domains. Female gender, older parental age, higher hyperactivity, and poorer prosocial behavior were associated with increased overall sleep disturbances.	Absence of a control group; no evaluation of sleep parameters by objective measures.
Hirata et al. (112) Japan	Case–control study	965 community; 193 preschoolers with ASD	*DSM-5* diagnostic criteria; ADOS-G; CBCL.	Japanese Sleep Questionnaire for Preschoolers.	Preschoolers with ASD had more sleep problems, including OSA, than those in the community, sleep problems, especially insomnia, correlated with behavioral problems in preschoolers with ASD.	No evaluation of sleep parameters by objective measures.
Hundley et al. (113) New York	Cross-sectional study	532 children with ASD (2–17 years).	*DSM-IV-TR* diagnostic criteria; ADOS; ADI-R; CBCL.	CSHQ.	Repetitive sensor/motor behaviors were positively associated with parent-reported sleep problems, and the relationship remained significant after controlling for anxiety symptoms. Insistence on sameness was not significantly associated with sleep problems.	No evaluation of sleep parameters by objective measures; absence of a control group.
Irwanto et al. (114) Indonesia and Japan	Cross-sectional study	50 children with ASD.	*DSM-5* diagnostic criteria.	CSHQ.	There were significant differences in total night and early awakenings between Indonesian and Japanese children.	Small sample size; absence of a control group. No evaluation of sleep parameters by objective measures.
Fletcher et al. (115) England	Case–control study	21 children with ASD; 29 TD children.	*DSM-IV-TR* diagnostic criteria; Spence Children’s Anxiety Scale; Social Worries Questionnaire; Bedtime Routines Questionnaire	CSHQ; 14 nights of actigraphy	There was a significant reduction in sleep duration over time in both groups, and the ASD one showed more night-to-night variability in sleep quality. Reductions in actigraphy-derived sleep efficiency were associated with an increased frequency of maladaptive activities in the hour before bedtime, in children with and without ASD.	Small sample size.
Mazurek et al. (116) New York	Cross-sectional study	81 children with ASD.	Physical Aggression and the Hostility subscales of the Children’s Scale for Hostility and Aggression: Reactive/Proactive (C-SHARP); the Inattention and the Hyperactivity subscales of the Vanderbilt Attention Deficit/Hyperactivity Disorder Parent Rating Scale (VADPRS).	CSHQ.	Sleep problems were significantly associated with physical aggression, irritability, inattention, and hyperactivity. Night awakenings had the most consistently strong association with daytime behavior problems, even after controlling for the effects of age and sex.	No evaluation of sleep parameters by standardized measure.
Kheirouri et al. (117) Turkey	Case–control study	35 children with autism; 31 TD subjects	*DSM-IV-TR* diagnostic criteria.	CSHQ.	There was no significant association between GI problems and autism severity, but a significant positive correlation have been found between different indicators of sleep disorders and severity of autism. Plasma levels of serotonin were significantly high in autistic children, but no significant association with sleep problems.	Small sample size; no evaluation of sleep parameters by standardized measure.
Mutluer et al. (118) Turkey	Case–control study	64 patients with ASD; 53 TD subjects.	*DSM-IV-TR* diagnostic criteria; CARS; CBCL.	Pediatric sleep questionnaire.	Children with ASD had higher frequency of sleep problems, snoring, breathing problems, behavioral problems compared with healthy children; sleep latency was prolonged in children with ASD compared with healthy subjects.	No evaluation of sleep parameters by standardized measures; no gender and other confounding factors examination.
Veatch et al. (119) USA and Canada	Cross-sectional	80 children with autism and sleep onset delay (2–10 years).	*DSM-IV-TR* diagnosis criteria; ADOS.	CSHQ; actigraphy.	Reported problems with sleep onset delay were concurrent with sleep duration problems in 66% of children, night wakings in 72% of children, and bedtime resistance in 66% of children; 38% of children reported insomnia. Parent reports and actigraphy results were in accordance.	Relatively small sample size.	
Aathira et al. (120) New Delhi India	Longitudinal study	71 children with ASD; 65 TD children.	*DSM IV* diagnostic criteria; CARS; DP-3 (Developmental Profile-3).	PSG (48 subjects); CBCL.	The prevalence of poor sleepers among ASD and controls were 77.5% and 29.2%, respectively. The salient findings on PSG were reduced sleep efficiency, decreased REM and SWS duration 1. The CBCL score was significantly high in poor sleepers compared to good sleepers on CSHQ. There was no correlation of CARS or DP-3 score with sleep problems in ASD children.	Small sample size; PSG only for a night.	
Kose et al. (121) Turkey	Retrospective cross-sectional study	48 children with ASD; 46 children with ID; 48 TD children (2–18 years).	*DSM-5* diagnostic criteria; CARS; Wechsler Intelligence Scale for Children-Revised (WISC-R); Ankara Developmental Screening Inventory (ADSI).	CSHQ.	Children with NDD had a 2.8-fold increased risk of sleep disturbance, 3.1-fold increased risk for history of sleep disorders in parents, 3.3-fold increased risk for psychiatric comorbidity, 13.1-fold increased risk for co-sleeping with parents. Co-sleeping with parents and family history of sleep problems increased the risk of sleep disturbances.	No evaluation of sleep parameters by standardized measures; Turkish adaptations of ADI-R and ADOS are not yet available.	
Sannar et al. (122) USA	Cross-sectional study	106 hospitalized children and adolescents with ASD (9.5–16.3 years).	Aberrant Behavior Checklist-Community (ABC-C); ADOS-2.	Sleep habits.	High scores on the ABC-C (irritability, stereotypy, and hyperactivity subscales) at admission were significantly associated with fewer minutes slept during the last five nights of hospitalization. There was no association between total awakenings and ABC-C scores or ADOS-2 comparison scores.	No evaluation of sleep parameters by standardized measures; absence of a control group.	
Veatch et al. (123) USA	Cross-sectional	2714 children in the Simons Simplex Collection.	*DSM-5* diagnostic criteria; ADI-R; ADOS; Differential Ability Scales, 2nd Edition; Mullen Scales of Early Learning.	Parental report of sleep; actigraphy; CBCL.	Sleep duration and severity of core ASD symptoms were negatively correlated, sleep duration and IQ scores were positively correlated. Severe social impairment was strongly associated with short sleep duration. Increased severity for numerous maladaptive behaviors, as well as reports of attention deficit disorder, depressive disorder, and obsessive compulsive disorder were associated with short sleep duration. Severity scores for social/communication impairment and restricted and repetitive behaviors were increased, IQ scores were decreased in children who reported to sleep 420 min per night compared to children sleeping 660 min.	No evaluation of sleep parameters by standardized measures; wide age range; use of medication.	
Verhoeff et al. (124) Netherlands	Longitudinal study	5151 children (81 children with ASD)	*DSM IV/5* diagnostic criteria; Pervasive Developmental Problems score (PDP) of the CBCL; Social Responsiveness Scale (SRS).	Sleep Problem Scale.	Sleep problems in early childhood were prospectively associated with a higher SRS score, but not when correcting for baseline PDP score. A higher SRS score and an ASD diagnosis were associated with more sleep problems at later ages, even when adjusting for baseline sleep problems. A trajectory of increasing sleep problems was associated with ASD.	No evaluation of sleep parameters by standardized measures; absence of a control group.	

DSM, Diagnostic and Statistical Manual; ICD-10, International Classification of Diseases-10; ASD, autism spectrum disorder; AS, Asperger syndrome; HFA, high-functioning autism; PDD, pervasive developmental disorders; DD, developmental delay; CSHQ, Children’s Sleep Habits Questionnaire; CARS, Childhood Autism Rating Scale; ASSQ, The High-Functioning Autism Spectrum Screening Questionnaire; ADI, Autism Diagnostic Interview; ADOS, Autism Diagnostic Observation Schedules; PSG, polysomnography; CBCL, child behavior checklist; IQ, intelligence quotient; TD, typically development; WHO, World Health Organization; REM, rapid eye movement.

Researches based on parental reports and objective measurements (actigraphy and polysomnography) have suggested that autistic children are more likely to have sleep difficulties than children with other neurodevelopment disorder (69, 70, 74, 75, 78, 80, 83, 90, 107) and children with normal development (62, 64, 69, 80, 85, 90, 91, 120). Among autistic subjects, some studies have reported that children with intellectual disabilities and/or with low levels of functioning may experience higher frequency of sleep disturbances (58, 62), while other studies have reported that high-functioning autistics may experience more sleep problems (shorter sleep duration, early night awakenings, and longer sleep latencies) than low-functioning children and control groups (63, 64). Some studies conducted in adolescents have also reported that those with high-functioning autism were three times more likely to have sleep problems than their peers with normal development (102). The most frequent sleep pattern observed across all the studies included the following: difficulties falling asleep (63–65, 72, 78, 89, 91, 109), restless sleep (64, 72), difficulties falling asleep in own beds with late bedtime (58, 104, 119), frequent nighttime awakenings (58, 63, 65, 70, 72, 78, 85, 91, 104, 107, 119), early morning awakenings (63, 65, 72), low sleep efficiency (85, 91), short time duration (58, 63, 72, 109, 104, 119), and daytime sleeplessness (71, 109). Nightmares, morning headaches, crying during sleep, sleep apnea (72), and sleepwalking (64, 72) were also frequent. Although sleep problems were significant across all ages, subjects during adolescence tend to show a shorter sleep duration than toddlers who tend to present more frequent bedtime resistance, sleep anxiety, parasomnias, and night awakenings (98, 115). Some of the studies reviewed have analyzed sleep disturbances by means of the polysomnographic registrations in autistic subjects. Studies have shown a significant reduction of REM sleep proportion and a significant presence of REM activity in non-REM sleep compared to subjects with normal development (66, 69). One study (66) on 10 autistic children and 8 children with Down Syndrome reported sleep continuity was disturbed by an increased number of night awakenings with a consequent reduction of sleep efficiency. In a later study (68), patients with AS showed a decrease in sleep time in the first two-thirds of the night and an increase in number of shifts into REM sleep from night awakenings. As reported in other studies, subjects with ASD may experience prolonged sleep latency, decreased sleep efficiency, and decreased sleep duration on the first night of polysomnographic registration (80, 93, 120). Studies based on actigraphic registrations confirmed the data recorded using logs and/or sleep diaries observing frequent early night arousals, a delay of sleep period, insomnia, and less total sleep time within the 24 h in autistic children (67, 86, 87, 90, 126). As reported in several studies, insomnia and sleepiness were correlated to the severity and prevalence of typical autism behavioral symptoms (69, 71, 73, 76, 77, 83, 84, 95, 99, 100, 112, 113). In particular, a study of Schreck et al. (73) found a correlation between insomnia and the prevalence of communication problems due to an increased sensitivity to stimuli in the sleeping environment. Further studies (101, 111) found a correlation between typical autistic behaviors and chronic insomnia. Children with ASD and poor sleep were characterized by a major representation of symptoms of autism such as restricted/repetitive behaviors (91, 113), language impairment, problems with reciprocal social interactions, low overall intelligence (80, 100, 103, 109), and even physical aggression, irritability, and oppositional defiant disorder (83, 84, 116, 122). Affective disturbances, particularly depression (80) and anxiety-related problems (71, 95, 103, 105, 109), have even been described in ASD with poor sleep. Some research groups (97, 100, 106) have analyzed the relation between insomnia, psychopathology, and daytime functioning in both children and adolescents with autism and reported a severity of depressed mood, anxiety, and daytime functioning in relation to sleep disturbances. Rzepecka and colleagues (95) reported a significant correlation between sleep problems, challenging behavior, and anxiety in children with intellectual disability and/or ASD. This study indicated that the 41.9% of the variance of challenging behavior was predicted by sleep problems and anxiety. The first study (110) that has analyzed the relationship between anxiety, sensory problems, and sleep disturbances in autism showed that children with anxiety and sensory overresponsivity may be particularly predisposed to develop sleep problems. A recent study of Veatch et al. (123) has demonstrated that shorter sleep duration was associated not only with depressive and obsessive–compulsive disorders but also with social impairment such as a failure to develop peer relationships and with maladaptive behaviors in ASD. Children who reported 7 h of sleep per night showed a severe impairment in social/communication compared to ASD children who tended to sleep 10–11 h per night (123). Anxiety, autism symptom severity, sensory sensitivities, snoring, obstructive sleep apneas, pain, and gastrointestinal problems were also associated with sleep disturbances (103, 108, 112, 118). In summary, sleep disturbances are frequent in children and adolescents with ASD. Insomnia and sleepiness were the most commonly reported symptoms together with long sleep latency, long time spent awake after sleep onset, early time awakening, and short sleep time. As shown in several studies, sleep disturbances have been related to the severity of ASD symptoms such as difficulties in social interaction and communication, presence of stereotypic behaviors, as well as anxiety and depressive symptoms.

### Circadian Sleep Dysrhythmicity in Adults With Autism Spectrum Disorder

Five studies analyzed the circadian rhythmicity in adults with ASD (**Table 3**). The most frequent circadian sleep disorders was the delayed sleep phase (126, 128–130). The most recent study (130) has investigated two groups (41 adults with ASD and intellectual disability versus 51 normal development) by means of ambulatory circadian monitoring, recording temperature, motor activity, body position, sleep, and light intensity; the circadian phase advanced was more common in the ASD group compared to controls. In summary, despite the fact that a few studies have investigated circadian sleep dysrhythmicity in adults with ASD, all the results showed that a delayed sleep phase is frequent in adults with ASD.

**Table 3:** Clinical studies on circadian rhythmicity and autism spectrum disorder in adults.

Authors (Ref.) country	Study design	Sample size	ASD assessment	Sleep–wake cycle assessment	Results	Major limitations
Hare et al. (126) Manchester, UK	Comparative study	10 adults with AS; 18 TD adults.	ICD-10 or *DSM-IV* diagnostic criteria; ASQ.	Actigraphy; basic sleep diary	Adults with AS showed significant phase advancement in the sleep–wake cycle with longer sleep latency, lower sleep efficiency, and more fragmented sleep than TD ones.	Small sample size; participants recruited from a relatively small area; need for more closely matched participants.
Hare et al. (127) Manchester, UK	Comparative study	31 adults with ID, of whom 14 had an ASD.	IQ score; *DSM IIIR/IV* diagnostic criteria; ASSQ; British Picture Vocabulary Scale (BPVS)	Actigraphy; basic sleep diary	No significant differences in sleep quantity and quality between the participants depending on whether they had an ASD.	Small sample; participants recruited from a relatively small area; use of medication; people with unidentified ASD symptoms in the non-autistic group.
Baker and Richdale (128) Australia	Cross-sectional study	36 adults with ASD; 36 TD adults.	AQ; ADOS-2.	14-day sleep–wake diary, 14-day actigraphy assessment; Composite Scale of Morningness questionnaire.	Delayed sleep–wake phase disorder, advanced sleep–wake phase disorder, and non-24-h sleep–wake rhythm disorder were present in the participants. A higher proportion of adults with ASD met criteria for a circadian rhythm sleep–wake disorder (CRSWD) compared to control adults delayed sleep–wake phase disorder.	Small sample size; no evaluation of factors that contribute to CRSWDs.
Goldman et al. (129) USA	Comparative study	28 adolescents/young adults with ASD; 13 age/sex matched TD individuals (11–26 years).	*DSM-IV-TR* diagnostic criteria; ADOS.	Adolescent Sleep–Wake Scale; Adolescent Sleep-Hygiene Scale; 4 weeks actigraphy; melatonin salivary collections (over 4 nights, starting at 6:00 pm and repeated every 30 min until bedtime); salivary cortisol collected immediately before bedtime and immediately upon awakening for 4 days starting on the morning after the last night of melatonin sample collection.	Compared to those with TD, adolescents/young adults with ASD had longer sleep latencies and more difficulty going to bed and falling asleep. Morning cortisol, evening cortisol, and the morning–evening difference in cortisol did not differ by diagnosis (ASD vs. TD). Dim light melatonin onsets (DLMOs) averaged across participants were not different for the ASD and TD participants.	Small sample size; emphasized HFA; participants with erratic sleep schedules, a broad age range and taking medications; not define circadian preference; psychiatric comorbidities.
Ballester et al. (130) Spain	Case–control study	41 adults with ASD and ID (<70); 51 TD adults.	*DSM-5* diagnostic criteria.	Ambulatory circadian monitoring (ACM) device has three different sensors: Wrist temperature, actimeter information (motor activity and body position), light intensity; 7-day sleep–wake diary.	Circadian phase advance in the ASD group was suggested by the higher values for wrist temperature and sleep and the lower motor activity and body position during the late afternoon and the first part of the night when compared to controls. Individuals with ASD and ID presented sleep difficulties (low sleep efficiency, prolonged sleep latency and increased number and length of night awakenings), together with daily sedentary behavior, increased nocturnal activity and a consistent phase advance in circadian rhythms.	Small sample size; ACM has not been validated in ASD to study sleep; most ASD were medicated; the group with ASD was not matched with the control group on sex, IQ, employment status, or living conditions.

DSM, Diagnostic and Statistical Manual; TD, typically development; ID, intellectual disabilities; IQ, intelligence quotient; ICD-10, International Classification of Diseases-10; ASD, autism spectrum disorder; AS, Asperger syndrome; HFA, high-functioning autism; ADOS, Autism Diagnostic Observation Schedules; ASQ, Autism Screening Questionnaire; AQ, autism quotient.

### Sleep Disturbances in Adults With Autism Spectrum Disorder

To date, only nine studies have described the sleep pattern on adults with ASD (**Table 4**). Three studies conducted from the same group (131–133) that compared AS to controls have shown similar polysomnographic sleep patterns. On the other hand, some further studies have suggested that 80% of adolescents and young adults with autism and AS may experience sleep problems (68, 106, 135). Sleep disturbances described in adults with ASD were similar to those described in children and were characterized by low sleep efficiency, short sleep duration, long sleep latency, frequent nighttime awakenings, and daytime sleepiness (135, 137). High-functioning adults with ASD reported insomnia and/or a significant phase advancement showing longer sleep latency, more frequent nocturnal awakenings, lower sleep efficiency, increased duration of NREM stage 1, and decreased non-REM slow-wave sleep compared to the healthy control group (134). Further, low total sleep time was correlated with social and communication impairments in autistic subjects (106, 134). Some authors have also reported that the presence of poor sleep in adults with high-functioning autism correlated with various aspects of motor output on nonverbal performance tasks, suggesting that sleep disturbances in ASD might affect attention and/or memory components in the nonverbal modality (136). In summary, the main sleep problems in adults with ASD were insomnia, low sleep efficiency, short sleep duration, long sleep latency, frequent nighttime awakenings, and daytime sleepiness. As shown in some studies, sleep disturbances were related to difficulties in social interaction, communication, and cognitive performance.

**Table 4:** Clinical studies on sleep disturbances in autism spectrum disorder in adults.

Authors (Ref.) country	Study design	Sample size	ASD assessment	Sleep–wake cycle assessment	Results	Major limitations
Godbout et al. (68) Canada	Case–control study	8 patients with AS (7–53 years); 8 age-/gender-matched TD subjects.	*DSM-IV* diagnostic criteria; ADI.	Sleep was recorded for two consecutive nights and scored according to standard methods using 20-s epochs	Patients with AS showed decreased sleep time in the first two-thirds of the night, increased number of shifts into REM sleep from a waking epoch, and all but one patient showed signs of REM sleep disruption. EEG sleep spindles were significantly decreased while K complexes and REM sleep rapid eye movements were normal. Three patients with AS, but none of the comparison participants, showed a pathological index of periodic leg movements in sleep.	Small sample size; use of medications.
Tani et al. (131) Helsinki, Finland	Case–control study	20 AS patients; 10 TD subjects.	*DSM III-R* diagnostic criteria; Beck Depression Inventory; Wechsler adult intelligence scale, revised version; ASSQ.	Basic Nordic Sleep Questionnaire; 6-day sleep diary.	AS adults reported frequent insomnia in all measures.	Small sample size.
Tani et al. (132) Helsinki, Finland	Case–control study	20 adults with insomnia (19.9–34.5 years); 10 age-/gender-/education-matched TD subjects.	*DSM-IV-TR* diagnostic criteria.	2-nights PSG. Results of the second night recordings were included in the analysis.	AS subjects displayed a similar PSG profile compared with controls. Sleep periods were equal in both groups with a great amount of slow-wave sleep in the early part of the night. The only sign indicating decreased sleep continuity in autistic subjects was the greater proportion of wake after sleep onset.	Small sample size.
Tani et al. (133) Finland	Longitudinal study	20 adults with AS; 10 age-/sex-/intelligence-matched TD adults.	Clinical diagnosis.	Actigraphy.	People with AS did not differ from the controls regarding actigraphic sleep profiles.	Small sample size.
Limoges et al. (134) Canada	Cross-sectional study	27 adults with HFA (16–27 years); 78 TD subjects (16–30 years).	*DSM-IV* diagnostic criteria; ADI-R; Wechsler Adult Intelligence Scale 3ed.	Sleep habits questionnaire; Horne and Os¨tberg’s questionnaire to determine morningness–eveningness typology; laboratory sleep recordings for two consecutive nights; Achenbach Youth Self-Report scale to measure of adaptive behaviors; State–Trait Anxiety Inventory; Beck Depression Inventory; Cortisol saliva samples.	Autism group: a longer sleep latency, more frequent nocturnal awakenings, lower sleep efficiency, increased duration of stage 1 sleep, decreased non-REM sleep and slow-wave sleep, fewer stage 2 EEG sleep spindles, and a lower number of rapid eye movements during REM sleep vs. TD participants; no differences between group on the Beck Depression Inventory; trait anxiety scores on the Spielberger Anxiety Scale were higher in ASDs. Objective total sleep time correlated negatively with the Social and Communication scales of the ADI-R. The sleep structure of clinical subgroups did not differ, except fewer EEG sleep spindles in the Asperger syndrome subgroup.	Small sample size.
Oyane and Bjorvatn (135) Bergen, Norway	Cross-sectional study	9 adolescents and young adults with autistic disorder; 6 adolescents and young adults with AS; (15–25 years)	Clinical diagnosis.	Sleep questionnaire; Epworth Sleepiness Scale; 2 weeks sleep diaries; 2 weeks actigraphy.	Although the sleep questionnaires completed by parents revealed only a moderate degree of sleep problems, great sleep disturbances were recorded with actigraphy. Low sleep efficiency (below 85%) or long sleep latency (more than 30 min) have been found in 80% of the subjects. There was no early morning awakening.	Small sample size; absence of a control group.
Limoges et al. (136) Canada	Case–control study	17 adults with ASD (9 with HFA and 8 with AS); 14 TD individuals.	*DSM-IV* diagnostic criteria. ADI-R; a battery of nonverbal tasks was administered, in the morning after a second night of sleep in the laboratory.	PSG.	Signs of poor sleep in the autism group were significantly correlated with either normal performance (selective attention and declarative memory) or inferior performance than controls (sensory-motor and cognitive procedural memories). Both groups presented a significant negative correlation between slow-wave sleep and learning a sensory-motor procedural memory task.	Small sample size; large number of correlation.
Richdale et al. (106) Australia	Case–control study	27 adolescents with HFASD (14.2–16.8 years); 27 age-sex-matched TD; adolescents (14.4–16.6 years).	The Centre for Epidemiological Studies Depression scale (CES-D); the anxiety subscale of the Depression, Anxiety and Stress Scale (DASS-21)	7-day sleep/wake diary; actigraphy; Sleep Habits Survey; Chronic Sleep Reduction Questionnaire to measure daytime functioning; Sleep Anticipatory Anxiety Questionnaire to measure pre-sleep arousal.	HFASD group reported significantly higher scores for depressed mood, anxiety and pre-sleep arousal vs. TD adolescents, and poorer daytime functioning. More significant correlations between sleep variables and psychopathology variables, and sleep variables and daytime functioning in the HFASD group, vs. TD group.	Small sample size.
Baker and Richdale (137) Australia	Cross-sectional study	36 adults with HFASD; 36 age-/sex-/intelligence quotient-matched TD adults.	Autism Quotient; ADOS-2.	Online questionnaire battery: PSQI; 14 days sleep diary; 14 days actigraphy.	HFASD group reported significantly more general sleep disturbances, high scores on the PSQI, long sleep onset latencies (actigraphy), and poor sleep efficiency (diary) and these results remained significant after accounting for the false discovery rate. HFASD group reported significantly shorter total sleep time, poorer refreshment scores upon waking in the morning and higher scores on the daytime dysfunction due to sleepiness subscale of the PSQI compared to the TD group.	Small sample size; use of medications.

DSM, Diagnostic and Statistical Manual; ASD, autism spectrum disorder; AS, Asperger syndrome; HFA, high-functioning autism; ADI, Autism Diagnostic Interview; ADOS, Autism Diagnostic Observation Schedules; PSG, polysomnography; TD, typically development; REM, rapid eye movement; PSQI, Pittsburgh Sleep Quality Index.

### Melatonin and Autism Spectrum Disorder

According to our research methods, we found 17 studies on the role of melatonin in circadian rhythm dysregulation in ASD (**Table 5**). Two different studies (138, 139) measured melatonin serum levels every 4 h for 24 h in autistic subjects compared to healthy controls. They found an abnormal melatonin circadian rhythm, low blood concentration during the night related to sleep disturbances, and a tendency to have high daytime melatonin levels in autistic subjects. A later study by Tordjman and colleagues (140) analyzes the overnight urinary excretion of the predominant melatonin metabolite, 6-sulfatoxymelatonin (6-SM). Sixty-three percent of post-pubertal subjects with ASD showed low 6-SM, most marked in males and prepubertal children; this finding was confirmed in a later study (145). Another research group (146) confirmed a reduction in urinary secretion of 6-SM in subjects with ASD. The previous results were confirmed in another study, and low nocturnal excretion of 6-SM was associated with a greater severity of autistic symptoms (verbal language and repetitive behaviors). In a study of Pagan et al., (150), patients with ASD showed higher serotonin and lower melatonin levels than healthy controls. Patients with melatonin deficit reported more frequent sleep-onset and sleep-maintenance insomnia than patients with normal melatonin levels. The same research group investigated, for the first time, melatonin synthesis in the pineal gland and in the gut of patients with ASD, reinforcing the hypothesis that the nocturnal increase in circulating melatonin was reduced in those subjects and caused by enzymatic disruption of both aralkylamine *N*-acetyltransferase (AANAT) and acetylserotonin *O*-methyltransferase (ASMT) involved in melatonin synthesis confirming a melatonin reduction (153). An Afghanistan study (152) confirmed a reduction of melatonin serum level that was related to the severity of ASD symptoms. Recently, melatonin salivary concentrations have been studied in ASD subjects with and without anxiety and/or depression compared to controls (154). The timing of the dim light melatonin onset (DLMO) did not differ between the two groups, but advances and delays of the melatonin rhythms were the circadian sleep disorder more frequently observed in ASD subjects. Regarding the genetic pathways of melatonin, several studies focused on the variations of genes that regulate the synthesis, metabolism, and mechanism of action of melatonin. A multicenter study, the Paris Autism Research International (Sib-pair study) (141), sequenced all ASMT exons and promoters in 250 individuals with ASD. The ASD group showed non-conservative variations of the protein sequence of ASMT, and only two ASD families presented a splicing mutation. Forty-three individuals with ASD revealed a highly significant decrease of ASMT activity and melatonin level compared to 48 controls. Another subsequent research from the Sib-pair study (142) analyzed 941 individuals: 295 patients with ASD, 362 controls, and 284 individuals from different ethnic backgrounds. They sequenced MTNR1A and MTNR1B (melatonin receptor 1A and 1B) genes coding for melatonin 1 (MT1) and melatonin 2 (MT2) receptors, and G protein-coupled receptor 50 (GPR50) gene, coding for the orphan melatonin-related receptor GPR50. Authors found MTNR1A and MTNR1B non-synonymous mutations altering the functional properties of the human melatonin receptors. Regarding GPR50, they detected a significant association between ASD and two variations in affected males. In the same year, Sweden researchers (143) screened the genetic mutations of AANAT, ASMT, MTNR1A, MTNR1B, and GPR50, encoding both synthesis enzymes and the three main receptors of melatonin, in 109 patients with ASD and 188 controls. In this study, several rare variants were found in melatonin-related genes in patients with ASD, including the mutation in ASMT. A Chinese study (147) investigated all ASMT exons and the neighboring region in 398 individuals with ASD and 437 healthy controls. They detected new rare coding mutations of ASMT affecting the protein sequence only in six individuals of the ASD group, but the authors did not find significant differences of genotypic distribution and allele frequencies of the common SNPs in ASMT between groups. Only one study (148) investigated a large sample of 1,747 subjects by means of the analysis of the SNPs and the duplication of exons 2–8 in ASMT, in order to identify genes involved in autism psychopathology. The authors identified the association between one SNP in the last intron of ASMT and social interaction impairments in females, but they did not detect any relation with language impairment or restricted and repetitive behaviors. According to previous research, Veatch et al. (151) studied 15 ASD children with sleep disturbances, of whom 11 were treated with melatonin, and found that an association between sleep onset delay and dysfunctional variation in genes related to the melatonin pathway, especially with regard to cytochrome (CYP) 1A2. They also, observed a strong correlation between genotypes in ASMT and in CYP1A2, particularly in the subset of children who responded to treatment with supplemental melatonin. In summary, most of the studies that focused on autistic children have found abnormal melatonin circadian secretion and low blood and salivary melatonin concentrations during the night with a tendency to have high daytime melatonin levels. Regarding the genetic pathways of melatonin, several studies focused on the variations of genes that regulate the synthesis, metabolism, and mechanism of action of melatonin (AANAT, ASMT, MTNR1A, MTNR1B, and GPR50) showing several rare genetic variants of them. These findings have been related to sleep disturbances in ASD.

**Table 5:** Clinical studies on melatonin in autism spectrum disorder.

Authors (Ref.) country	Study design	Sample size	ASD assessment	Sleep–wake cycle assessment	Melatonin (MT)	Results	Major limitations
Nir et al. (138) Jerusalem, Israel	Case–control study	10 males with ASD (16–30 years); 5 TD subjects.	*DSM-III* diagnostic criteria.		Blood melatonin level every 4 h for 24 h.	Abnormal melatonin circadian rhythm in autistic; amplitude of melatonin peak lower in children with autism than in controls; serum melatonin higher during day and lower during night than in controls.	Small size sample. No evaluation of sleep parameters.
Kulman et al. (139) Italy	Case–control study	14 children with autism; 20 age-matched TD children.	*DSM-III* diagnostic criteria.		Blood melatonin level every 4 h for 24 h.	Significantly low melatonin level in autistic; abnormal melatonin circadian rhythm in all 14 autistic children compared with controls: children with ASD did not demonstrate physiological increase in melatonin during the night.	Small size sample; no objective sleep measures. No evaluation of sleep parameters.
Tordjman et al. (140)	Case–control study	49 children and adolescents with autistic disorder; 88 TD children matched on age, sex, and Tanner stage of puberty (6–15 years).	*DSM-IV*, ICD-10, and CFTME diagnostic criteria; ADI-R; ADOS-G; Wechsler intelligence scales; Kaufman-Assessment Battery for Children (K-ABC)		Urinary 6-SM 12 h collection from 8 pm to 8 am.	Mean 6-SM lower than in controls; 63% of children with ASD had low 6-SM, low 6-SM level was significantly more common in males and prepubertal children.	No diurnal melatonin evaluation. No evaluation of sleep parameters.
Melke J et al. (141) Paris Autism Research International Sipair study	Case–control study; multicentric study	250 autistic patients and their parents; 255 TD subjects.	*DSM-IV* diagnostic criteria; ADI-R; Social and Communication Disorders (DISCO-10); AS Diagnostic Interview.		Sequencing ASMT exons and promoters; biochemical analyses performed on blood platelets and/or cultured cells.	Non-conservative variations of ASMT including a splicing mutation present in two families with ASD, but not in controls. Two polymorphisms located in the promoter (rs4446909 and rs5989681) were more frequent in ASD compared to control and were associated with a decrease in ASMT transcripts in blood cell lines. Highly significant decrease in ASMT activity and melatonin level in individuals with ASD.	No evaluation of sleep parameters.
Chaste P et al. (142) Paris Autism Research International Sib-pair study	Case–control study; multicentric study	295 patients with ASD (AD = 222; AS = 61; PDD-NOS = 12); 362 TD subjects; 284 individuals from different ethnic backgrounds.	*DSM-IV* diagnostic criteria; ADI-R; AS Diagnostic Interview.	Actigraphy; analysis of MT1-I49N mutation.	Sequenced MTNR1A, MTNR1B, and GPR50 genes (coding for the orphan melatonin-related receptor GPR50) in patients and controls.	6 non-synonymous mutations for MTNR1A and 10 for MTNR1B. Most of these variations altered receptor function. Mutants are MT1-I49N, which is devoid of any melatonin binding and cell surface expression, and MT1-G166E and MT1-I212T, which showed severely impaired cell surface expression. The prevalence of these deleterious mutations in cases and controls indicates that they do not represent a major risk factor for ASD (MTNR1A case 3.6% vs. controls 4.4%; MTNR1B case 4.7% vs. 3% controls). They detected a significant association between ASD and two variations OF GPR50, D502–505 and T532A, in affected males.	
Jonsson et al. (143) Sweden	Case–control study	109 patients with ASD; 188 TD subjects.	*DSM-IV* diagnostic criteria; ADI-R.		They have investigated all the genes involved in the melatonin pathway by mutation screening of AA-NAT, ASMT, MTNR1A, MTNR1B, and GPR50.	Several rare variants were identified in patients with ASD, including splice site mutation in ASMT (IVS5+2T > C). However, mutations were found in upstream regulatory regions in three of the genes investigated, ASMT, MTNR1A, and MTNR1B.	No evaluation of sleep parameters.
Mulder et al. (144) Netherlands	Case–control study	10 normoserotonemic and 10 hyperserotonemic age-matched autistic individuals.	*DSM-IV-TR* diagnostic criteria: ADI-R; ADOS.		Urinary excretion of 5-hydroxyindoleacetic acid (5-HIAA) and serotonin (5-HT).	In the hyperserotonemic group, significant increases at trend level in urinary excretion of 5-HIAA and 5-HT and a significant decrease for 6-SM were found. The urinary 5-HIAA:5-HT ratio was similar in the normoserotonemic versus the hyperserotonemic groups.	No characterization of daytime and nighttime melatonin production in autism. No evaluation of sleep parameters.
Leu et al. (145), Nashville, USA	Cross-sectional study	23 children with ASD (4–10 years)	ADOS; ADI-R; Peabody Picture Vocabulary Test-III (PPVT-III); the Parental Concerns Questionnaire (PCQ).	CSHQ; PSG.	Overnight Urinary 6-SM.	Urinary 6-SM excretion rates are low in autistic subjects. Higher urinary 6-SM excretion rates were associated with increased N3 phase of sleep, decreased N2 phase of sleep, and daytime sleepiness.	Small sample size; lack of a control group; did not obtain information on the specific segments of sleep; no repeated blood specimens of melatonin.
Tordjman et al. (146) France	Case–control study	Post-pubertal individuals with autism (*N* = 43) and TD controls (*N* = 26)	*DSM-IV-TR*, ICD-10 diagnostic criteria; ADI-R; Wechsler intelligence scale; Kaufman K-ABC		Daytime and nighttime urinary excretion of 6-SM during a 24-h period.	Low 6-SM excretion in autism; nocturnal excretion of 6-SM was negatively correlated with autism severity in the overall level of verbal language and repetitive use of objects.	No evaluation of sleep parameters.
Wang et al. (147) Beijing, Republic of China	Case–control study	398 patients with autism (2–17 years); 437 healthy controls.	*DSM-IV* diagnostic criteria; Autism Behavior Checklist (ABC); CARS.		They sequenced all ASMT exons and their neighboring region.	The study did not detect significant differences of genotypic distribution and allele frequencies of the common SNPs in ASMT between patients with autism and healthy controls; new rare coding mutations of ASMT.	Small sample size; lack of clinical information; not sequence other genes in melatonin pathway.
Jonsson et al. (148) Sweden	Cross-sectional study	1747 subjects (357 monozygotic (MZ) twin pairs, 500 dizygotic (DZ) twin pairs, and 33 subjects without their co-twin).	Telephonic interview: The Autism—Tics, ADHD and other Comorbidities is a sensitive tool for screening the general population for child ASDs and associated conditions.		Analysis of the SNPs and the duplication of exons 2–8 in ASMT. A panel of 47 SNPs to determine twin zygosity.	Significant association, in girls, between an intronic SNP and social interaction impairment and restricted and repetitive behavior where the C-allele carriers were shown to have higher scores. They also investigated a microduplication of exons 2–8 in the ASMT gene, which was found in 27 individuals (1.7%). All these individuals had one extra copy of the region investigated, except for one MZ twin pair, who had two extra copies. This duplication was analyzed with respect to the total ASD scores, although no significant associations could be shown.	Small sample size; no evaluation of sleep parameters.
Goldman et al. (149) Nashville, USA	Cross-sectional study	9 children (3–10 years) and took at least 30 min or longer at baseline to fall asleep on three or more nights (by parent report and actigraphy), free of psychotropic medication.	*DSM-IV-TR* diagnostic criteria; ADOS.	Comprehensive sleep interview; video-EEG-PSG; actigraphy; CSHQ.	Endogenous plasma melatonin dim light melatonin onset (DLMO) and supplemental melatonin.	In endogenous samples, maximal melatonin concentration and time to peak concentration were comparable to the literature results for TD children. DLMO were captured in the majority of children. Children with ASD and insomnia responsive to low dose melatonin treatment have relatively normal profiles of endogenous and supplemental melatonin.	Small sample size, lack of a control group; variability in the specific start time of the serial blood sampling.
Pagan et al. (150) Paris Autism Research International Sib-pair study	Case–control study; multicentric study	278 patients with ASD, their 506 first-degree relatives (129 unaffected siblings, 199 mothers and 178 fathers); 416 sex- and age-matched controls.	Social Responsiveness Scale (SRS), in first-degree relatives and in controls; RBS (Repetitive Behavior Scale) for probands and their relatives; ADI-R; Diagnostic Interview for Genetic Studies for adults and Kiddie-Schedule for Affective Disorders and Schizophrenia for children; Wechsler scales or Raven’s progressive matrices for nonverbal individuals.	Sleep self-report and/or parent questionnaire; CSHQ; actigraphy; Pittsburgh Sleep Quality Index; a self-assessment questionnaire to determine morningness–eveningness in human circadian rhythms; Epworth sleepiness scale.	Whole-blood serotonin was measured by high-performance liquid chromatography. Plasma melatonin was measured using a radioimmunoassay. NAS and 14-3-3 were determined in platelet pellets; urine samples were collected overnight (2000–0800 hours) from 16 adult patients with HFA and 10 adult controls; 6-SM was measured by a radio immunological method.	Patients showed higher serotonin and NAS levels and lower melatonin levels than healthy controls. Impairments of melatonin synthesis in ASD may be linked with decreased 14-3-3 proteins. The melatonin deficit was only significantly associated with insomnia.	The assessment of melatonin only from plasma sampled in the morning; the association finding was not fully replicated in an independent study.
Veatch et al. (151) Nashville, USA	Cross-sectional	15 ASD children (3–9 years) with sleep disturbances of which 11 in treatment with melatonin.	*DSM-IV-TR* diagnostic criteria; ADOS.	Sleep interview followed by structured parent education to provide instructions on daytime and evening habits to promote sleep. Children were confirmed to have sleep onset delay of at least 30 min at baseline on C3 nights per week, and none had sleep disturbance limited to specific seasons.	They evaluated variation in two melatonin pathway genes, ASMT and cytochrome P450 1A2 (CYP1A2).	Higher frequencies than currently reported for variants evidenced to decrease ASMT expression and related to decreased CYP1A2 enzyme activity relationship between genotypes in ASMT and CYP1A2	Lack of a control group; small size sample; unable to assess potential differences in ASMT and CYP1A2 between responders and non-responder to melatonin.
Abdulamir et al. (152) Baghdad, Iraq	Case–control study	60 males with ASD (3–13 years) divided into 3 subgroups: mild, moderate, and severe; 26 TD subjects age-/gender- matched.	*DSM-5* diagnostic criteria.	76% of autistic subjects showed sleep problems. The severe autistic patients showed the highest number of sleep problems (18 patients) in comparison with moderate (15 patients), and mild autistic patients (13 patients).	Serum levels of melatonin and oxytocin.	Levels of oxytocin and melatonin in patients were significantly lower than that of age-matched and gender-matched controls and were associated with the severity of the disease that was indicated by the significant decrease in the levels of oxytocin and melatonin in moderate patients.	Small sample size; only single area; no systematic assessment of sleep–wake rhythm.
Pagan et al. (153) France	Cross-sectional study	Melatonin: 9 patients and 22 controls; gut samples for serotonin: 11 patients and 13 controls; blood platelets: 239 individuals with ASD and their first-degree relatives and 278 controls.	*DSM-IV-TR* diagnostic criteria.		Melatonin in plasma and tissues was measured using a radioimmunoassay; serotonin was measured by high-performance liquid chromatography; NAS, AANAT, and ASMT were determined by radio enzymology; the amount of 14-3-3 proteins was determined using the commercial 14-3-3 Pro ELISA kit.	Melatonin deficit in ASD, reduction of AANAT and ASMT observed in the pineal gland as well as in gut and platelets of patients. Reduced levels of 14-3-3 proteins that regulate AANAT and ASMT activities and increased levels of miR-451.	Small samples size; no evaluation of sleep parameters.
Baker et al. (154) Australia	Case–control study	16 adults with ASD (ASD-Only); 12 adults with ASD medicated for comorbid diagnoses of anxiety and/or depression (ASD-Med); 32 TD subjects.	ADOS-2; Autism Quotient (AQ); Wechsler Abbreviated Scale of Intelligence (WASI); Wechsler Adult Intelligence Scale-Fourth Ed (WAIS IV): 3 ASD-Only.	14-day sleep/wake diary and actigraphy assessment; State–Trait Anxiety Inventory (STAI); Patient Health Questionnaire-8 (PHQ-8); Sleep Anticipatory Anxiety Questionnaire (SAAQ).	Sit in dim light 1-h prior to their first saliva sample with saliva sampling commencing 3 h prior to their habitual sleep time and ceasing 1 h past their habitual sleep time, with an hourly sampling rate; salivary melatonin concentrations were determined by a commercially available Melatonin EIA kit.	The timing of DLMO did not differ between the two groups, advances and delays of the melatonin rhythm were observed in individual profiles. Overall mean melatonin levels were lower in the ASD-Med group compared to the two other groups; greater increases in melatonin in the hour prior to sleep were associated with greater sleep efficiency in the ASD groups.	Small sample sizes; use of the individual saliva collection protocols to assess DLMO in adults with ASD; inability to measure and control participants’ exposure to blue light.

DSM, Diagnostic and Statistical Manual; ICD-10, International Classification of Diseases-10; ASD, autism spectrum disorder; AD, autism disorder; AS, Asperger disorder; ADHD, attention-deficit hyperactivity disorder; PDD-NOS, pervasive developmental disorders–not otherwise specified; TD, typically development; CSHQ, Children’s Sleep Habits Questionnaire; ADI_R, Autism Diagnostic Interview-Revised; ADOS, Autism Diagnostic Observation Schedules; CARS, Childhood Autism Rating Scale; PSG, polysomnography; CBCL, child behavior checklist; ASMT, acetylserotonin O-methyltransferase; MTNR1A, MTNR1B, melatonin receptor 1A and 1B; GPR50, G protein-coupled receptor 50; AA-NAT, arylalkylamine N-acetyltransferase; 6-SM, 6-sulfatoxymelatonin.

## Discussion

This systematic review aimed to provide the current status of knowledge about sleep disturbances and circadian sleep disorders in ASD across the lifespan. The current data have shown a number of several striking findings with regard to circadian sleep rhythmicity and ASD, but the nature of their link remains unclear. The literature on adult population is scant compared to studies on children. Although few studies have investigated this topic in adult subjects, similar results have been found compared to children. As shown in our results, studies have reported a high frequency of sleep problems and alterations of circadian sleep rhythmicity in ASD across all ages (55, 65, 71, 95, 108, 109, 114, 125). Difficulties in falling asleep, frequent nighttime awakenings, and short sleep duration were the sleep disturbances most frequently described in both children and adults with ASD (72, 78, 79, 124, 130). Accordingly, these results were also confirmed by means of actigraphic and polysomnographic registration, displaying specific irregular sleep–wake cycles, low sleep efficiency, long sleep latency, insomnia, daytime sleepiness (68, 91, 97, 106, 119, 120, 132, 133, 134, 136, 137), delayed circadian phases, and evening preference as chronotype associated with ASD. According to our searches, polymorphisms in CLOCK genes that regulate sleep may be associated with ASD (25, 26, 59, 60). Evidence is not strong although there are four separate studies implicating the alteration in CLOCK gene expression in the dysregulation of circadian sleep rhythmicity in ASD (25, 26, 59, 60). Melatonin dysregulation, which includes delay in melatonin peak, reduction in amplitude, and alteration in melatonin gene expression, may also contribute to circadian sleep desynchronization in ASD [for an overview, see Ref. (155)]. In some studies, blood, salivary, or urinary levels of melatonin or of its metabolites have been shown to be reduced in autistic subjects (138–140, 145, 150) and to be correlated directly or indirectly with severe autistic behaviors such as verbal communication as well as repetitive behaviors and daytime sleepiness (146, 152). According to our results, abnormalities in melatonin-related genes (enzymes involved in melatonin synthesis, metabolism and⁄or melatonin receptor function) may be related to low melatonin levels in ASD or may lead to an altered response to melatonin in a proportion of individuals with ASD (141–143, 148, 153).

**Figure 2:**
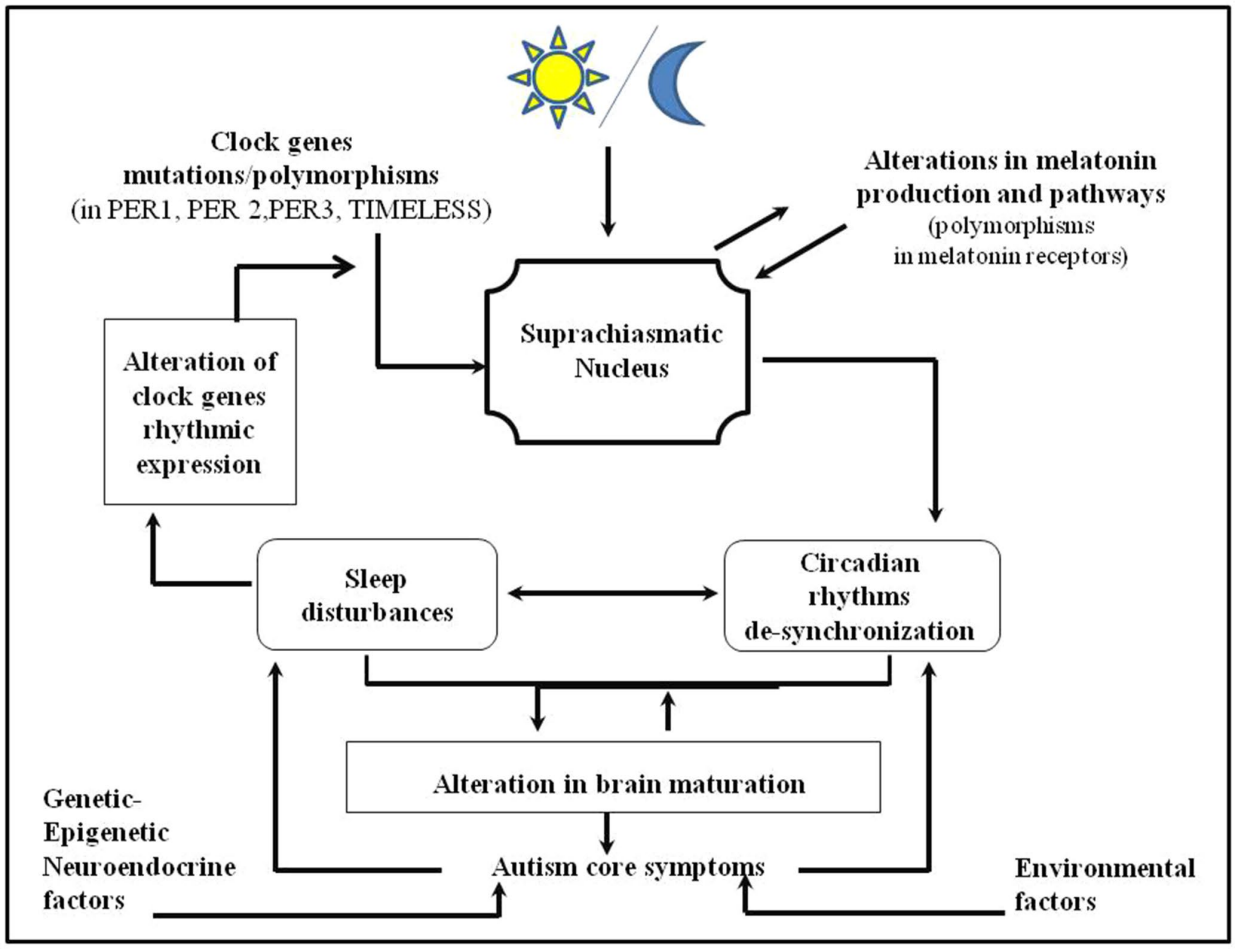
Integrative model of the relationship between autism spectrum disorders, circadian and sleep dysrhythmicity. Polymorphism in clock genes and alterations in melatonin pathways may contribute to alterations in circadian sleep rhythms and consequently in the sleep regulation in toto. Altered sleep may negatively influence the brain maturation contributing to the autism core symptomatology. Vice versa, autism symptomatology may reinforce sleep disturbances creating a self-reinforcing loop between them.

Several studies pointed out the potential correlation between sleep disturbances and the severity of autistic symptoms above all repetitive behaviors and deficits in verbal communication and/or in social reciprocity (73, 100, 103, 109, 113, 156, 157). In summary the current data have shown several striking findings with regard to sleep/circadian sleep function and ASD, but the nature of the link remains unclear. We may hypothesize that polymorphisms in clock genes and alterations in melatonin pathways may contribute to the dysregulation of the circadian sleep rhythmicity and consequently to the dysregulation of sleep system *in toto*. According to the theories about the functions of sleep during development (30, 36–40), we may hypothesize that sleep disturbances may negatively influence brain maturation, contributing to autism symptoms. It has already been hypothesized that even a modest and initial impairment of circadian sleep rhythmicity may increase the individual’s vulnerability to ASD [for an overview, see Ref. (11)]. Indeed, on the other hand, autistic symptoms may reinforce sleep disturbances, creating a self-reinforcing feedback loop (**Figure 2**). This framework should be useful in order to identify elements to evaluate and target in the clinical practice. In particular, sleep disturbances and circadian sleep alterations may represent a novel therapeutic target in ASD. According to this model, by treating sleep and circadian sleep disorders in ASD, we should contribute to an improvement in ASD symptoms. There is strong evidence about the use of melatonin in ASD, indicating its beneficial effects on sleep and autistic symptoms. As suggested by some studies (158–165), melatonin may improve sleep difficulties in ASD subjects even when administered in association with concurrent psychotropic medications (160) and/or cognitive behavior therapy (164). Treatment with melatonin seems to improve sleep disturbances in the majority of children and adults with autistic symptoms (166), showing effectiveness on sleep duration, sleep latency, and nocturnal and early morning awakenings (158, 159, 161–165, 167, 168). Most importantly, by treating sleep and circadian sleep disorders with melatonin, an improvement in typical autistic behaviors has been shown in some studies (158, 163, 168). Data in children with neurodevelopmental disorders failed to point to serious adverse events associated with the use of melatonin (160, 162, 153, 165, 169) except for morning sleepiness (165), headache, and fatigue (167). These findings shed light on a rising hypothesis of an integrative model that may explain the interference of circadian rhythms and its variables during critical periods of brain development in ASD (**Figure 2**). Evaluating and targeting sleep disturbances and circadian sleep disorders in ASD should be useful to improve the trajectory of ASD. Indeed, the current data have shown a “research gap” about this topic. Future research should focus on the systematic study of circadian sleep dysrhythmicity in children and adults with ASD in relation to clock gene expression and to melatonin production.

## Limitations

Our findings should be considered in the light of several limitations. Primarily, although this review aimed to summarize the most relevant studies on the relation between circadian sleep disorders and ASD, the inclusion/exclusion of specific studies may reflect our individual point of view or expertise and training. Moreover, some studies included may have been underpowered (some had only small sample sizes and small numbers of subjects enrolled) and/or some of them did not include control groups. Another limitation was the heterogeneity of the studies regarding the characteristics of the sample such as age, scholarity, study design, diagnostic criteria and measures of assessment, severity of sleep, and autistic symptoms that are potentially very important and should be considered routinely in future studies. Since the studies included in our research cover a wide temporal range (1984–2019), it is necessary to consider the nosography evolution of neurodevelopmental disorders over time, which could be a further study limitation. Psychiatric comorbidity and/or the use of psychopharmacological drugs may interfere with the duration and severity of both autistic symptoms and sleep disturbances. In addition, the potential roles of inert brain anomalies, the potential impact of brain injury during childbirth, and the potential influence of adverse environmental stressors in the early development of the child need to be addressed in further studies as being potential reasons for an apparent rise in the prevalence of ASD symptoms with later developmental stages.

## Conclusion

Overall, results from this systematic review highlight the idea that sleep and circadian sleep disturbances are frequent in subjects with autistic symptoms who have shown polymorphisms in clock gene expression and in genes involved in melatonin production. The impairment of circadian sleep regulation may increase the individual’s vulnerability to develop symptoms of ASD by impairing the sleep regulation *in toto*, which instead plays a key role in normal brain development. Even though controversies and “research gaps” are present in literature at this point, we may hypothesize a bidirectional relation between circadian sleep dysfunction and ASD. In particular, circadian sleep dysrhythmicity may predispose to develop ASD symptoms and vice versa within a self-reinforcing loop. An early identification and assessment of circadian sleep dysrhythmicity could be useful for improving treatment strategies in both children and adults with ASD.

## Author Contributions

LP, CC, LD’O, DC, and IM designed the study and finalized the article. IM and DC managed the literature search. IM and DC elaborated the PubMed results and developed the first draft of the manuscript. LP and CC reviewed the manuscript. LP, CC, AV, LN, and LD’O revised the final version. All authors contributed to and approved the final manuscript.

## Conflict of Interest Statement

The authors declare that the research was conducted in the absence of any commercial or financial relationships that could be construed as a potential conflict of interest.
